# Light-intensity switch enabled nonsynchronous growth of fluorinated raspberry-like nanoparticles†

**DOI:** 10.1039/d0sc04141f

**Published:** 2020-09-10

**Authors:** Shantao Han, Yu Gu, Mingyu Ma, Mao Chen

**Affiliations:** State Key Laboratory of Molecular Engineering of Polymers, Department of Macromolecular Science, Fudan University Shanghai 200433 China chenmao@fudan.edu.cn http://polymaolab.com/

## Abstract

Raspberry-like (RB) nanoparticles hold potential for diverse applications due to their hierarchical morphology. Here we developed a novel tandem synthetic approach of nonsynchronous growth based on photo-mediated reversible-deactivation radical polymerization, enabling simple, efficient and bottom-up synthesis of RB nanoparticles of uniform sizes at quantitative conversions of fluorinated monomers. Chain transfer agents of different chain lengths, concentrations and chemical compositions were varied to tune the diameter of RB particles. Importantly, fluorinated RB nanoparticles obtained with this method allow facile post modifications *via* both covalent bond formation and intermolecular physical interactions without disrupting the RB morphology. The facile nature of this method and versatility of the obtained fluorinated RB materials open new opportunities for the development of functional materials using nanoparticles.

## Abstract 2

Nonsynchronous growth of raspberry-like (RB) nanoparticles in a one-pot and bottom-up fashion, enabling simple post-modification of RB colloids through both covalent bond formation and supramolecular interaction.

## Graphical abstract

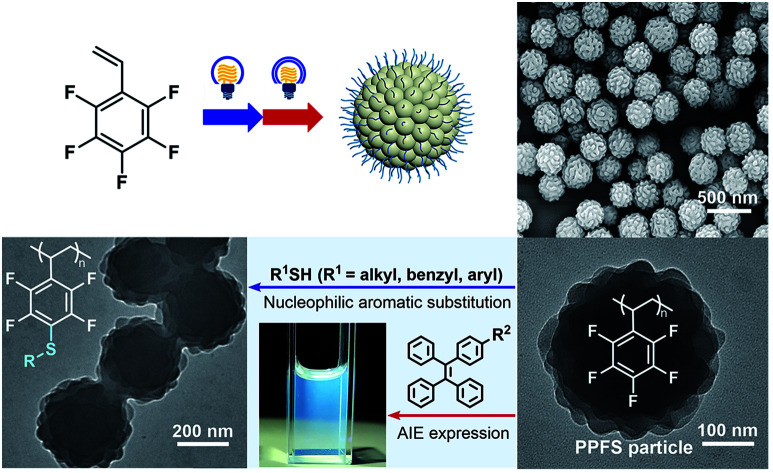

## Introduction

Nonspherical colloidal particles with anisotropic structures have been widely investigated due to their importance in fabricating functional materials.^1^ Among them, raspberry-like (RB) particles have gained considerable attention due to their surface roughness, large surface areas and high level of scattering ratios,^2,3^ leading to applications such as drug delivery,^4^ catalyst supports,^5^ photonic materials^6^ and superhydrophobic coatings.^7^ While reversible-deactivation radical polymerization (RDRP) has enabled the generation of polymeric particles with various morphologies^8–10^ ranging from spheres, worms, and vesicles to ordered inverse mesophases through the operationally simple polymerization induced self-assembly (PISA) pathway,^11–15^ it still remains challenging to generate RB particles based on PISA. Conventionally, RB particles have been obtained *via* the combination of small corona and large core particles *via* physical or chemical interactions,^16,17^ as well as emulsion polymerization.^18,19^ However, these strategies often suffer from complex operating processes or driving force of multiphase separation. Moreover, while post-synthetic modification represents an attractive approach to functionalize polymeric colloids, its utility for RB particles was confined due to lack of modifiable substituents and requirement of multistep processes.^20,21^ Therefore, the development of a facile PISA approach to prepare versatile RB particles would provide improved opportunities for materials engineering.

The employment of light as a low-cost and environmentally sustainable stimulus in RDRP^22–32^ (*i.e.*, photoinduced electron transfer–reversible addition-fragmentation chain-transfer (PET–RAFT) polymerization)^33–35^ has aroused a lot of interest, promoting the preparation of polymeric colloids without intermediate purification.^36–39^ Motivated by the outstanding physicochemical properties of fluoropolymers,^40,41^ we have developed photo-RDRP of fluorinated alkenes.^42,43^ While the fluorine–fluorine (F–F) interaction has been adopted to generate various morphologies,^44–47^ the preparation of fluorinated RB particles remains unexploited. Recently, kinetic mediation by light intensity in photo-RDRP has been demonstrated by Boyer^24^ and Miyake.^25^ We envisioned that by regulating the polymerization rate through visible light, self-assembly of fluorous substances would lead to nonsynchronous growth of successively-generated particles, resulting in polymeric colloids of different sizes, which would fuse to provide the RB morphology.

**Scheme 1:**
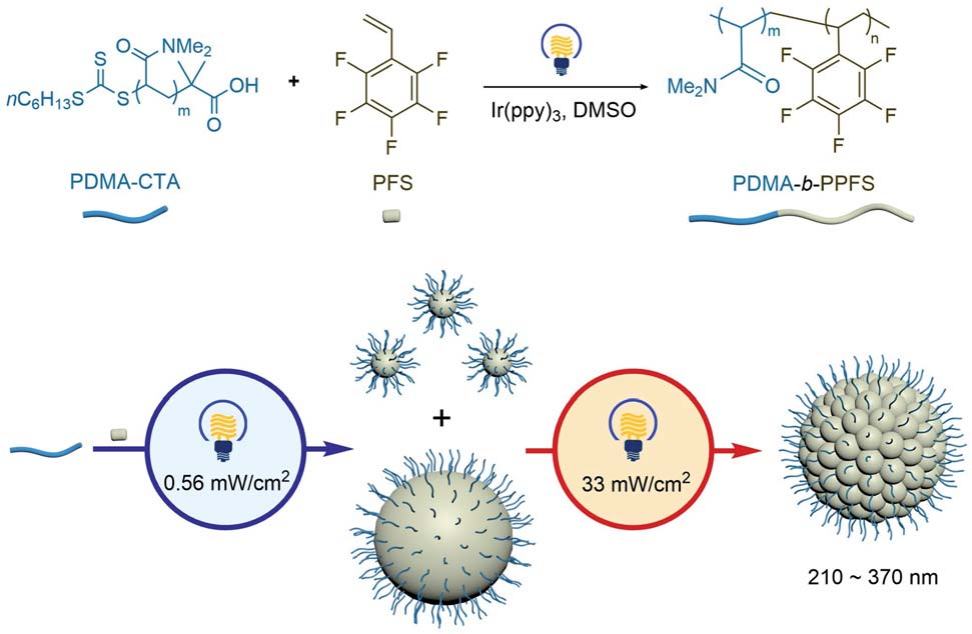
Synthesis of PPFS RB nanoparticles *via* a tandem photo-mediated approach based on photo-RDRP.

Herein, we report a one-pot, bottom-up synthesis of fluorinated RB particles through tandem photo-mediated nonsynchronous growth for the first time (Scheme 1), which represents a convenient and practical pathway to control the morphology of nanoparticles *via* the photo-RDRP of pentafluorostyrene (PFS). Moreover, the poly(PFS) (PPFS) backbone enables post modification of RB nanoparticles *via* both chemical and physical interactions, as demonstrated by cross-linking, substitution and aggregation-induced emission (AIE) experiments, providing an attractive platform for materials engineering.

## Results and discussion

At the beginning of our study, poly(dimethylacrylamide) (PDMA_54_, *M*_n_ = 5.64 × 10^3^ Da, *Đ* = 1.06) terminated with a trithiocarbonate group was prepared as a chain-transfer agent (CTA), and employed in the photo-RDRP of PFS using tris(2-phenylpyridine)iridium as a photocatalyst (PC) ([PFS]/[PDMA_54_-CTA]/[PC] = 300/1/0.1) under visible-light irradiation (400–750 nm, Fig. S1†) at room temperature. Dimethyl sulfoxide (DMSO) was adopted as solvent. In the formed copolymers, the PDMA block is solvophilic and the PPFS block is solvophobic. After optimization, light intensities of 33 and 0.56 mW cm^−2^ have been employed for the strong- and weak-light irradiations, respectively (Table S1†).

**Fig. 1:**
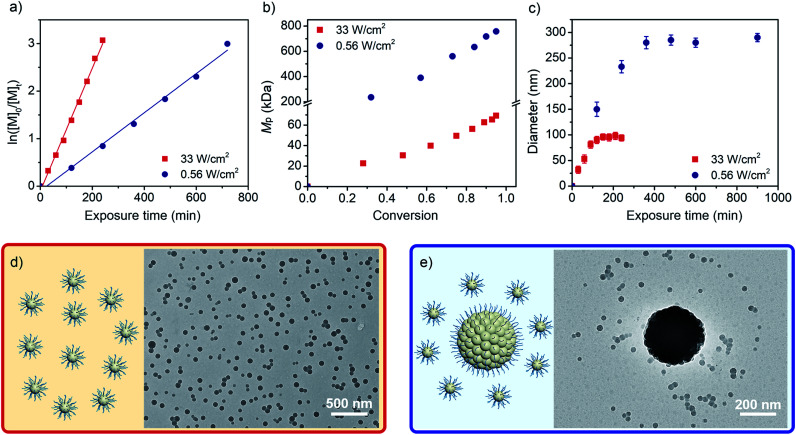
Preparation of PDMA_54_-*b*-PPFS_*n*_*via* photo-RDRP under exposure to a strong (33 mW cm^−2^) and weak light (0.56 mW cm^−2^). [PFS]/[PDMA_54_-CTA]/[PC] = 300 : 1 : 0.1. (a) Plots of ln([M]_0_/[M]_*t*_) *vs.* the exposure time, with [M]_0_ and [M]_*t*_ being the concentrations of PFS at time points 0 and *t*. (b) *M*_p_ determined by SEC *vs.* PFS conversion. (c) Particle diameter determined by DLS *vs.* PFS conversion. (d and e) Cartoon diagrams and TEM images of PPFS particles synthesized by exposure to strong- and weak-light irradiation. PFS conversions are 100% and 82%, respectively.

As shown in Fig. 1a, while the strong-light irradiation enables faster polymerization compared to weak-light (*k*_p_ = 0.77 *vs.* 0.25 h^−1^), both processes exhibit first-order kinetics during propagations. Although a two-stage regime of kinetics has been detected in PISA,^48,49^ our observations are consistent with RAFT polymerization of PFS under thermal conditions,^44^ where the absence of the first stage for homogeneous polymerization is attributed to the fast nucleation which occurred at very low degrees of polymerization (DPs) of the fluorinated core-forming block as driven by the F–F interaction.

The reaction mixtures were analyzed by size-exclusion chromatography (SEC). The peak molecular weight (*M*_p_) was adopted to indicate the changing trend of the molar mass of PPFS. The *M*_p_ values increase with PFS conversions under both strong- and weak-light irradiations (*M*_p_ = 22.7–69.1 *vs.* 235–746 kDa, Fig. 1b, S2, and S3, Tables S2 and S3†). The much higher *M*_p_ values observed for reaction mixtures under weak-light irradiation could be due to the low initiation efficiency of the CTA during the synthesis of the fluoropolymer. Under strong-light irradiation, more CTAs could be initiated, affording lower molecular weights at similar monomer conversions. As analyzed by dynamic light scattering (DLS, Fig. 1c and S4†), the diameters of particles first increase with the exposure time, and remain almost constant subsequently under both conditions. Meanwhile, decreasing the light intensity increases the final size of particles from about 92 to 318 nm. The nonlinear relationship between the colloidal volume and exposure time (Fig. S5†)^37^ suggests that a secondary nucleation could take place during the photo-RDRP process.

Next, the particles generated under both conditions were characterized by transmission electron microscopy (TEM). As shown in Fig. 1d, spheres of hydration diameter = 90 ± 10 nm were obtained using strong-light irradiation. Similarly, when other ratios of [PFS]/[PDMA_54_-CTA] (from 100/1 to 500/1) were employed to provide copolymers of different lengths of the fluorinated segment, only a spherical morphology was observed (diameter = 83 to 119 nm, *M*_p_ = 24.3–123 kDa, Fig. S6, S7 and Table S4†), which is different from the morphology evolution trend detected in other photo-PISA processes.^8,36^ In comparison, when weak-light intensity was used under otherwise identical conditions, large particles (341 nm) and a few small spheres (∼30 nm) have been simultaneously observed by TEM at 82% conversion of PFS (Fig. 1e).

Under weak-light irradiation, photoredox catalysis would generate relatively fewer propagating chains and lead to slower monomer consumption rates compared to strong-light irradiation. As a result, a portion of PDMA-CTA would first grow into fluorinated polymers and lead to the generation of fluorous particles *via* self-assembly. These particles would absorb PFS in solvent, subsequently provide fluoropolymers with high molar masses due to the increased molar ratio of the monomer to growing chain,^42^ and evolve into large particles during the relatively long-time polymerization. Meanwhile, the reaction between remaining PDMA-CTA and PFS dissolved in solvent would generate new fluorous particles of small sizes, which would have time to further fuse with large ones (Fig. S9†). In contrast, strong-light irradiation at the beginning of the reaction would promote the generation of more propagating chains, and enable high initiation efficiencies of PDMA-CTA (Fig. S2 and Table S2†). The formation of lots of fluorinated chains at the early stage would accelerate the formation of fluorous particles, which don't have enough time or remaining PFS to allow their growth into large ones.

Although the fusion of small and large particles could take place under weak-light irradiation, the remaining PFS absorbed in fused particles would act as plasticizers to increase the mobility of polymers.^45^ Before the complete consumption of PFS, the slow polymerization rate under weak-light irradiation could provide enough time for polymers to undergo chain movement, which would reduce the surface roughness of fused particles (Fig. S11†). We envisioned that accelerating the monomer consumption at the late-stage of polymerization could benefit maintaining the surface roughness.

**Fig. 2:**
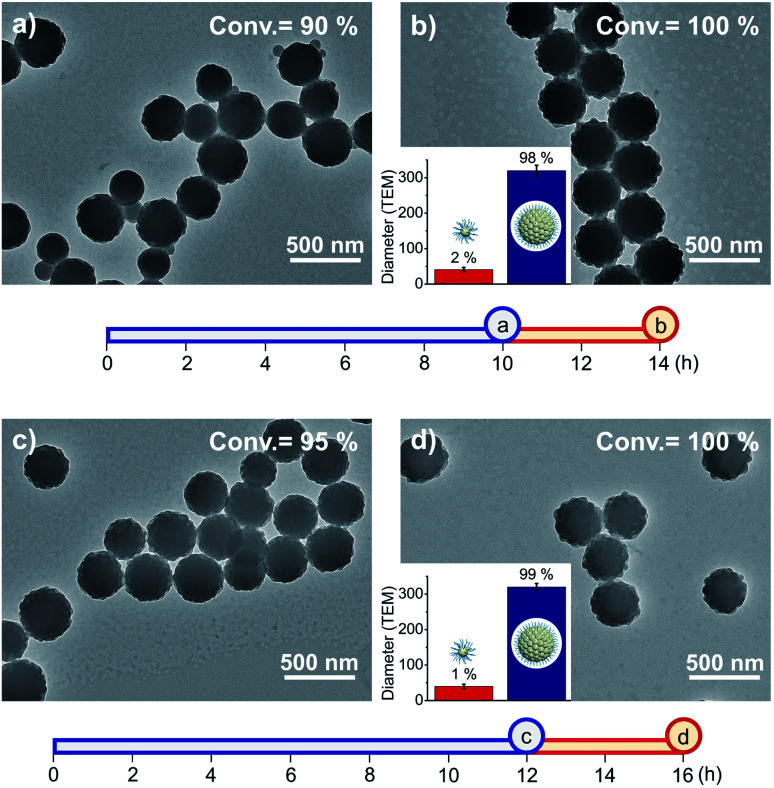
TEM images of fluorinated particles prepared by successive photo-RDRP using two light intensities. [PFS]/[PDMA_54_-CTA]/[PC] = 200 : 1 : 0.1. (a and c) Reaction mixtures were exposed to weak-light irradiation for 10 and 12 h, respectively. (b and d) Strong-light irradiation (4 h) was subsequently used following (a) and (c). The size and distribution of particles depicted in Fig. 2b and d were obtained by analysing approximately 400 random particles. The two axes exhibit the exposure times of two light intensities: blue colour for 0.56 mW cm^−2^, red colour for 33 mW cm^−2^.

Next, we attempted to apply weak- and strong-light irradiation successively to photo-RDRP in a one-pot fashion (Fig. 2a–d). After optimization of the switching timing (Fig. S10†), we found that when the light intensity was changed at high conversions (84–95%) of PFS, RB particles of uniform sizes were successfully obtained (327–341 nm, particle size distribution (PSD) = 1.05–1.14, Table S5†). During the reaction, formation and entanglement of fluoropolymers between different particles could stabilize the fused particles.^50^

According to the hypothesis, the fluorous cores should have higher PFS concentrations than that in solvent. Therefore, this method should lead to different populations of molar masses for polymers grown in fluorous cores and generated from CTA and PFS in solvents. When the reaction mixtures were analyzed by SEC, bimodal distributions were observed (*M*_p_ = 455–956 and 33.7–51.6 kDa, Fig. S8†), and the proportions of higher *M*_p_ values increased with the exposure time.

**Fig. 3:**
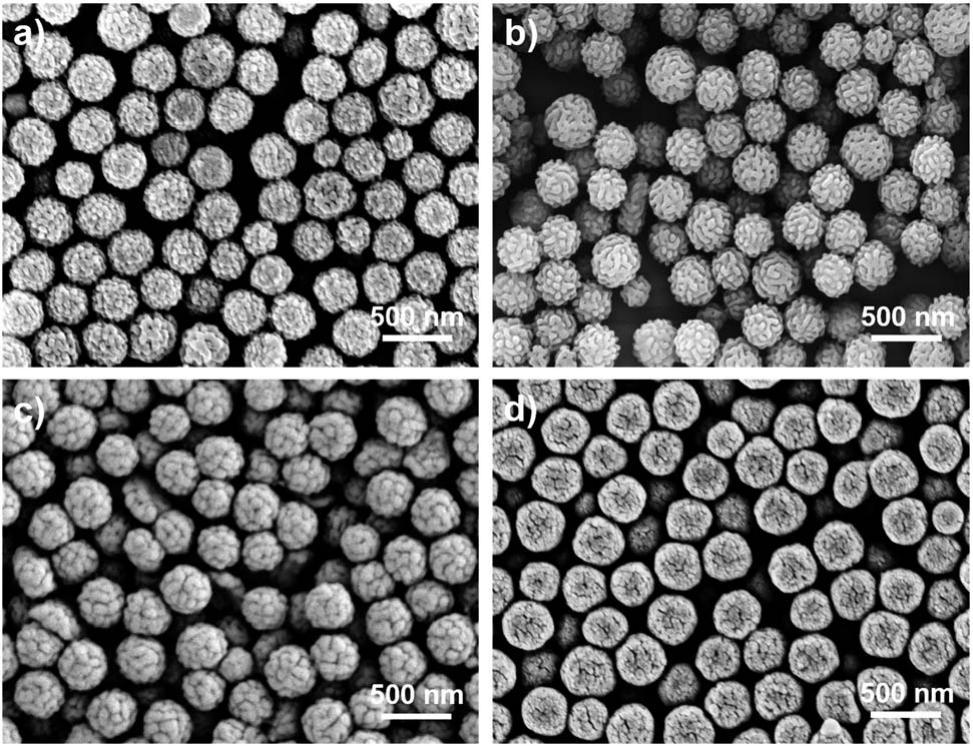
SEM images of RB particles synthesized at [PFS]/[PDMA_54_-CTA] = (a) 100/1, (b) 200/1, (c) 300/1, and (d) 500/1.

With the optimized method, RB nanoparticles were synthesized at a variety of ratios of [PFS]/[PDMA_54_-CTA] (100/1–500/1), and characterized by scanning electron microscopy (SEM). RB particles exhibited a narrow particle size distribution without purification (PSD = 1.05–1.28, *D*_h_ = 317 to 368 nm, Table 1, entries 1 to 4). The diameter of RB particles increased with the dosage of PFS relative to the CTA (Fig. 3), attributing to fluoropolymers of higher molar masses.^51,52^ When the DP of PDMA increased from 28 to 196 ([PFS]/[PDMA_*n*_-CTA] = 200/1), the diameter of RB particles decreased from 329 to 211 nm (PSD = 1.10–1.04, entries 2 and 5–7, Fig. S16†).^52^ When another macro-initiator (*i.e.*, polyethylene glycol (PEG) substituted CTA, entries 8) was employed, RB particles (*D*_h_ = 352 nm, PSD = 1.13, Fig. S12c†) were successfully generated, manifesting the versatility of this method. All particles in Table 1 exhibited bimodal molecular weight distributions indicated by SEC (Tables S6, and S7 and Fig. S13–S15†).

**Table 1:** Characterization of RB particles

Entry	CTA	[PFS]/[CTA]	*D*_h_ a (nm)	PSD^a^
1	PDMA_54_	100/1	317	1.28
2	PDMA_54_	200/1	332	1.07
3	PDMA_54_	300/1	347	1.07
4	PDMA_54_	500/1	368	1.05
5	PDMA_28_	200/1	329	1.10
6	PDMA_100_	200/1	276	1.08
7	PDMA_196_	200/1	211	1.04
8	PEG_113_^b^	200/1	352	1.13

a Hydration diameter (*D*_h_) and particle size distribution (PSD) of RB particles determined by DLS.

b CTA was substituted by PEG_113_.

**Fig. 4:**
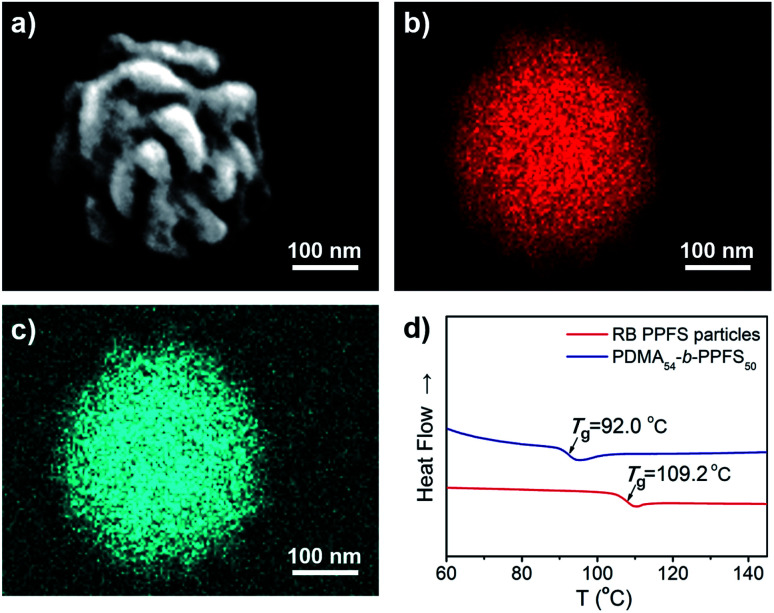
(a) SEM image of the RB particle. (b and c) Mapping images of elemental C and F. (d) DSC profiles of RB particles and PDMA_54_-*b*-PPFS_50_.

The obtained particles were characterized using an energy dispersive spectrometer (EDS, Fig. 4a). The EDS spectra exhibited a uniform distribution of C and F elements on the RB particles (Fig. 4b, c, S17 and Table S8†). The glass transition temperature (*T*_g_) of the RB particles (PDMA_54_-*b*-PPFS_4588_, DP of PFS was calculated based on *M*_p_, entry 2, Table S6†) was analyzed by differential scanning calorimetry (DSC, Fig. 4d), affording a *T*_g_ of 17 °C higher than that of PDMA_54_-*b*-PPFS_50_, owing to the increased molecular weight.

**Fig. 5:**
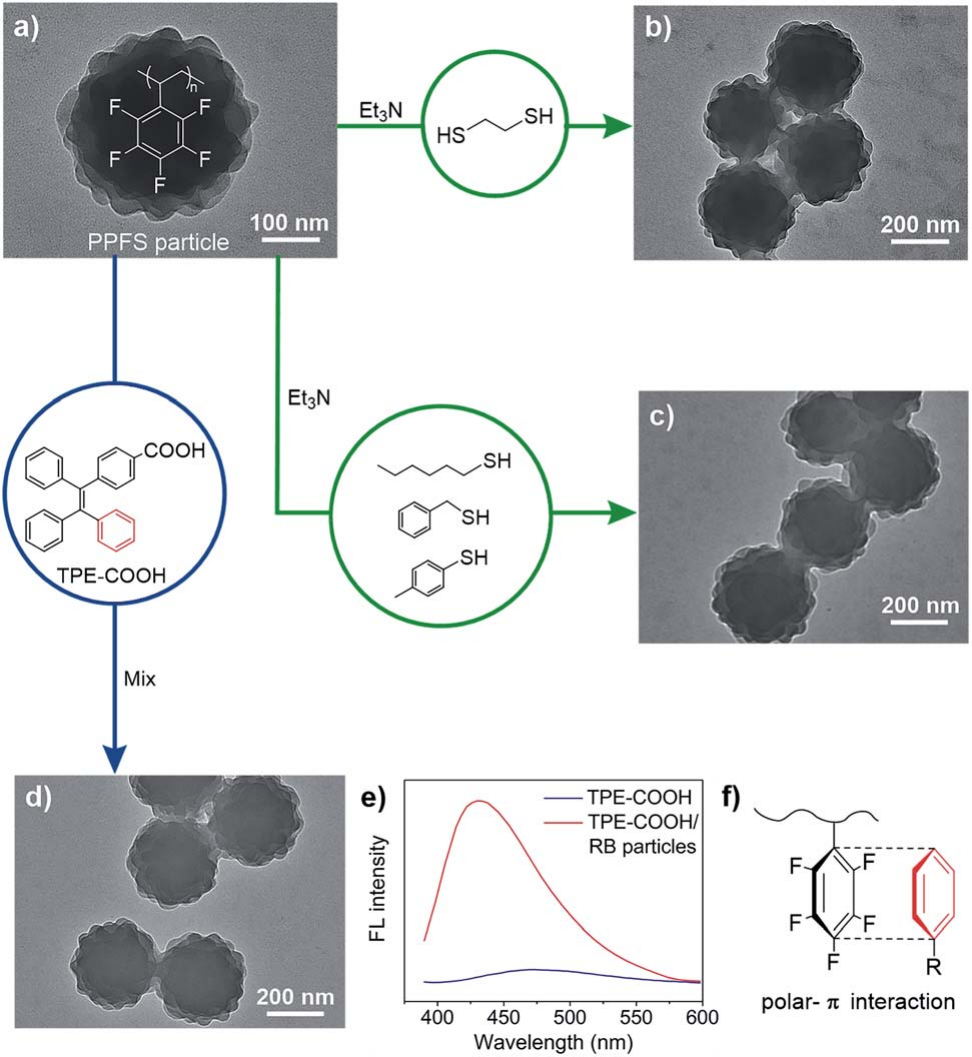
TEM images of RB particles before and after modification. (a) Original particles. (b) Treated with 1,2-ethanedithiol. (c) Treated with *p*-toluenethiol (hexanethiol and benzylthiol were used in Fig. S20†). (d) Treated with TPE-COOH. (e) Fluorescence profiles of TPE-COOH, and the mixture of TPE-COOH/RB particles. (f) Schematic illustration of polar-π interactions between –C_6_F_5_ of the particles and phenyl of TPE.

An advantage of PPFS is the ease in post-synthetic modification by nucleophilic aromatic substitution on the –C_6_F_5_ group, facilitating the preparation of functional polymers.^53,54^ In our studies, when RB particles were treated with 1,2-ethanedithiol, cross-linked colloids were generated without a clear morphological change (Fig. 5a and b). Compared to original RB particles, thermal stability of the rough surfaces of the cross-linked counterparts has dramatically increased for over 30 °C (80 *vs.* 115 °C, Fig. S20†).

Moreover, three types of nucleophiles including alkyl, benzyl and aryl thiols have been used to react with RB particles at room temperature. As determined by ^19^F nuclear magnetic resonance, the *para*-fluorine atom of the –C_6_F_5_ group could be efficiently converted in the nucleophilic aromatic substitution, while the RB morphology was maintained (Fig. 5c and Table S10†), furnishing opportunities for loading various molecules onto RB particles under mild and metal-free conditions *via* C–S bond formation.

Compounds with tetraphenylethylene (TPE) units show AIE behavior, and have stimulated biomedical and optoelectronic applications.^55^ Upon mixing a carboxyl substituted TPE (TPE-COOH) with RB particles, TPE-COOH was encapsulated into RB particles as indicated by the promoted AIE expression (Fig. 5d, e and S22†), exhibiting an enhanced fluorescence at 430 nm and a blue-shift of 40 nm compared to TPE-COOH due to restricted intramolecular rotation.^56,57^ The successful incorporation of TPE-COOH is probably due to the polar-π interaction^58,59^ between –C_6_F_5_ from the host particle and phenyl from the TPE guest (Fig. 5f).

## Conclusion

In conclusion, we have developed a tandem approach based on nonsynchronous growth *via* photo-RDRP that enables the one-pot and *in situ* synthesis of RB nanoparticles with uniform sizes at quantitative monomer conversion and under mild conditions. The successive visible-light irradiation from weak-to strong-light intensity has allowed the generation and fusion of large and small colloids, facilitating the synthesis of RB particles of a variety of sizes. Moreover, the fluorinated RB particles could be modified by both chemical and physical interactions, broadening opportunities to access fluorinated nanomaterials with various physicochemical properties. Given the broad interest in nonspherical colloids, fluorinated materials and photochemistry, we expect this method to be useful for providing versatile PPFS RB particles for advanced materials engineering.

## Conflicts of interest

There are no conflicts to declare.

## Supplementary Material

SC-011-D0SC04141F-s001
