# Supplementary material for: Light-intensity switch enabled nonsynchronous growth of fluorinated raspberry-like nanoparticles
Source: Chem Sci. 2020 Sep 10;11(38):10431–6. doi: 10.1039/d0sc04141f (PMC8162262; doi:10.1039/d0sc04141f)
Supplement: SC-011-D0SC04141F-s001 [file SC-011-D0SC04141F-s001.pdf]

Electronic Supplementary Material (ESI) for Chemical Science.
This journal is © The Royal Society of Chemistry 2020

Supplementary Information

Light-intensity switch enabled nonsynchronous growth of fluorinated raspberry-like nanoparticles

Shantao Han, Yu Gu, Mingyu Ma, Mao Chen*

State Key Laboratory of Molecular Engineering of Polymers, Department of Macromolecular Science, Fudan University, Shanghai 200433, China

*chenmao@fudan.edu.cn

Table of Contents

Section 1: **1**

1. General information of materials and analytical methods 1
2. Optimization of light intensity for the photo-RDRP of PFS 2
3. Investigation on the reaction kinetics for the photo-RDRP of PFS 3
4. Preparation of PPFS particles with different fluorinated lengths 7
5. Investigation on the formation of RB particles 8
6. Characterization of RB particles 16
7. Post-modification of fluorinated RB particles 17

Section 2: Other Supplementary Information **19**

1. Synthesis and characterization of PDMA-CTA 19
2. Synthesis and characterization of F-CTA 22
3. Synthesis and characterization of PEG_{113}-CTA 24
4. Synthesis and characterization of TPE-COOH 25

Reference **26**

Section 1:

1. General information of materials and analytical methods

2,3,4,5,6-Pentafluorostyrene (PFS) and N,N -dimethylacrylamide (DMA) were filtered through a basic aluminum oxide column to remove inhibitors before use. Dimethyl sulfoxide (DMSO), N,N-dimethylformamide (DMF) were freshly distilled from CaH2 before use. All the reagents were purchased from Sigma-Aldrich, Adamas or TCI, and were used as received without further purification.

Nuclear magnetic resonance (NMR) was conducted on an Advance III 400 MHz Bruker instrument at 298 K. Chemical shifts were measured according to the signal of residual chloroform (7.26 ppm) in deuterochloroform (CDCl3), and were reported in δ units (parts per million, ppm). Size-exclusion chromatography (SEC) measurements were performed in THF at 35 °C with a flow rate of 1.0 mL/min on an Agilent 1260 instrument equipped with a Waters 2410 refractive index detector. The calibration was performed with monodisperse poly(styrene) (PS) standards. Diameter of polymer particle and particle size distribution were measured by using a Zetasizer Nano (ZS90) from Malvern Instruments, Ltd. High Contrast Transmission Electron Microscope (HCTEM) was carried out on a Hitachi-7800 plus Microscope (Japan) at 120 kV. TEM samples were prepared by dropping 10 μL DMSO dispersion (0.1% w/v) on a carbon-coated copper grid and wiped by filter paper, then dried at room temperature for 48 h. Field-emission scanning electron microscopy and X - ray energy - dispersive spectroscopy (FESEM/EDS) was performed on a Zeiss Ultra 55 at 20 kV. Ethanol dispersions were diluted to 0.5% w/v and dropped onto silicon substrate that was attached to the steel stubs by carbon adhesive, and then dried under vacuum at 25 °C for 24 h. The SEM samples were sputtered a thin layer of gold to be conductive. Differential scanning calorimetry (DSC) was conducted on a TA Q2000 thermal analysis system at a scanning rate of 10 °C min-1 from 50 °C to 150 °C after eliminating the thermal history. The samples were prepared by centrifugation in ethanol and then further dried under vacuum at 30 °C for 24 h. The excitation and emission spectra of samples were recorded on an Edinburgh Instruments FLS1000 Transient/Steady-State Fluorescence Spectrometer. Emission spectra of LED light was tested by Ocean Optics

USB4000 Spectrometer and the corresponding software (Ocean View Spectroscopy Software). Column chromatography was carried out using silica gel (300-400 mesh).

2. Optimization of light intensity for the photo-RDRP of PFS

General experimental operation for Table S1:

An oven-dried 2 mL vial equipped with a stir bar was charged with 0.5 mmol monomer (PFS = 2,3,4,5,6-pentafluorostyrene), $PDMA_{54}$-CTA, tris(2-phenylpyridine)iridium (PC = $Ir(ppy)_3$) and 1 mL DMSO ([PFS]/[$PDMA_{54}$-CTA]/[PC] = 300/1/0.1). After the vial was sealed with a rubber septum, the solution was deoxygenated with three freeze-pump-thaw cycles under N_2 atmosphere. Then, the mixture was placed in front of a white LED light bulb at corresponding light intensity for 24 h while cooling with compressed air to maintain room temperature (25 °C). After reaction, internal standard (ethyl benzoate) was added into the mixture with stirring. A small aliquot was taken and analyzed by ^{1}H NMR to give monomer conversion (Conv. (%)) and DLS to give hydration diameter (D_h) and particle size distribution (*PSD*).

Table S1. Characterization results of $PDMA_{54}$-*b*-$PPFS_n$ synthesized by exposing to different light intensities.[a]

Entry	Power of light source (W)	Distance from the light source (cm)	Light intensity[b] (mW/cm^2)	Conv.[c] (%)	$M_{p,\,SEC}$ [d] (Da)	$Đ$ [d]	D_h [e] (nm) (*PSD*)
1	1	15	0.045	83	7.41×10^5	4.36	318 (1.32)
2	1	10	0.101	90	7.18×10^5	4.32	301 (1.25)
3	1	5	0.560	> 99	7.79×10^5	4.05	295 (1.10)
4	1	1	0.926	> 99	1.82×10^5	2.13	210 (1.12)
5	3	1	2.89	> 99	9.43×10^4	1.89	143 (1.07)
6	5	1	5.17	> 99	7.10×10^4	1.59	125 (1.06)
7	7	1	9.04	> 99	6.74×10^4	1.47	95 (1.09)
8	9	1	17.4	> 99	6.88×10^4	1.55	97 (1.06)
9	11	1	25.2	> 99	6.75×10^4	1.45	98 (1.10)
10	13	1	33.0	> 99	6.89×10^4	1.40	92 (1.05)

[a] [PFS]/[$PDMA_{54}$-CTA]/[PC] = 300:1:0.1, 10 wt % solid content, 24 h irradiation.

[b] Light intensity determined by the optical power meter of Thorlabs.

[c] Monomer conversion determined by ^1H NMR.

[d] Peak molecular weight (M_p) and molecule weight distribution ($Đ$) determined by SEC (THF solvent, PS standard).

[e] Hydration diameter of polymer particle (D_h) and particle size distribution (*PSD*) determined by DLS.

Based on results in Table S1, light intensities of 0.56 and 33.0 mW/cm^2 were chosen for the weak- and strong-light irradiation in following experiments, in order to provide full PFS conversion in short irradiation times and particles of big differences in diameters.

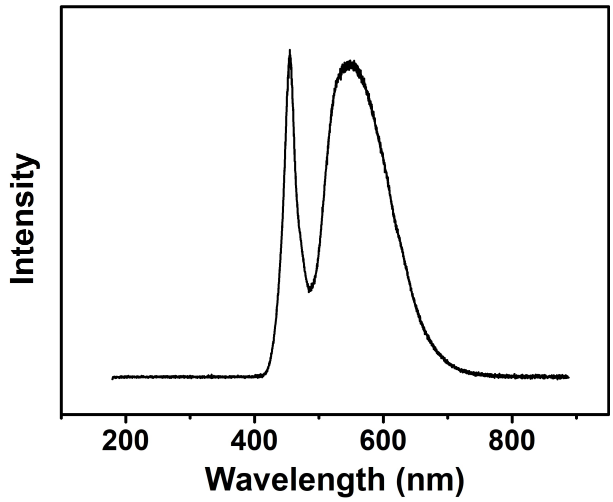

Figure S1 Emission spectrum of the white LED light bulb used to give strong- and weak-light irradiations.

3. Investigation on the reaction kinetics for the photo-RDRP of PFS

<u>*General experimental operation for Tables S2 and S3:*</u>

An oven-dried 4 mL vial equipped with a stir bar was charged with PFS (1.0 mmol), $PDMA_{54}$-CTA, $Ir(ppy)_3$, ethyl benzoate (1.0 mmol) and 2 mL DMSO ([PFS]/[$PDMA_{54}$-CTA]/[PC] = 300/1/0.1) in a glove box. The mixture was placed in front of a white LED light bulb while cooling with compressed air to maintain room temperature (25 °C). Small aliquots were taken at corresponding exposing times to give monomer conversions, particle diameters and TEM images by ^1H NMR, DLS and HCTEM measurements, respectively. Before analyzing by SEC instrument, samples were precipitated with ethanol for three times.

Table S2. Reaction results for the photo-RDRP of PFS with different exposing times under the light intensity of 33 mW/cm^2. [a]

Entry	Time (min)	Conv. [b] (%)	DP (PFS)	$M_{n, SEC}$ [c] (Da)	$M_{p, SEC}$ [c] (Da)	$M_{n, Th}$ [d] (Da)	$Đ$ [c]	D_h [e] (nm) (*PSD*)
1	30	28	84	2.01×10^4	2.27×10^4	2.16×10^4	1.43	32 (1.22)
2	60	48	144	2.95×10^4	3.05×10^4	3.33×10^4	1.27	53 (1.26)
3	90	62	186	3.65×10^4	3.98×10^4	4.14×10^4	1.29	81 (1.14)
4	120	75	225	4.79×10^4	4.95×10^4	4.90×10^4	1.37	90 (1.12)
5	150	83	249	5.46×10^4	5.62×10^4	5.36×10^4	1.28	96 (1.08)
6	180	89	267	5.98×10^4	6.27×10^4	5.71×10^4	1.32	95 (1.06)
7	240	95	285	6.32×10^4	6.91×10^4	6.06×10^4	1.43	98 (1.06)

[a] [PFS]/[$PDMA_{54}$-CTA]/[PC] = 300:1:0.1, 10 wt % solid content.

[b] Monomer conversion determined by ^{1}H NMR.

[c] Number-average molecular weight (M_n), M_p and $Đ$ determined by SEC (THF solvent, PS standard).

[d] Theoretical molecular weight = (target $DP_{(PFS)}$ × monomer conversion) × $M_{(PFS)}$ + DP × $M_{(DMA)}$ + $M_{(TTC)}$. $M_{(PFS)}$, $M_{(DMA)}$ and $M_{(TTC)}$ are molecular weights of PFS, DMA and the trithiocarbonate.

[e] D_h and *PSD* determined by DLS.

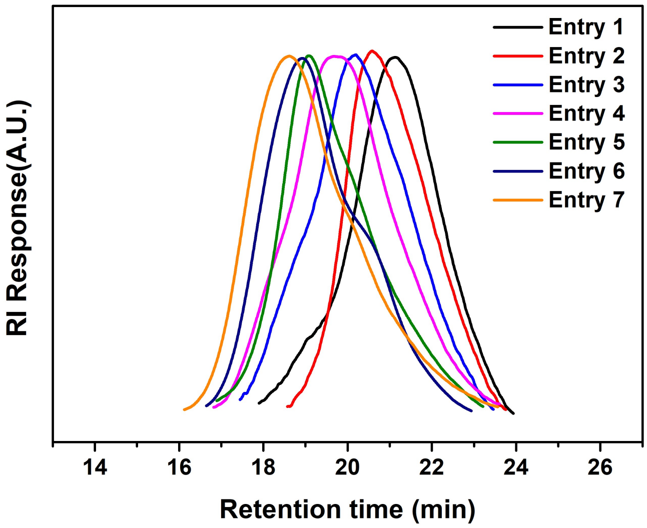

Figure S2 SEC profiles for reactions in Table S2 (light intensity = 33 mW/cm^2).

Table S3. Reaction results for photo-RDRP of PFS with different exposing times under the light intensity of 0.56 mW/cm^2.[a]

Entry	Time (min)	Conv. [b] (%)	$M_{p,\ SEC}$ [c] (Da)	$Đ$ [c]	D_h [d] (nm) (*PSD*)
1	120	32	2.35×10^5	3.23	150 (1.22)
2	240	57	3.90×10^5	5.26	233 (1.14)
3	360	73	5.61×10^5	4.25	278 (1.09)
4	480	84	6.34×10^5	4.41	285 (1.10)
5	600	90	7.17×10^5	4.17	280 (1.11)
6	720	95	7.58×10^5	3.94	290 (1.06)
7	1440	> 99	7.46×10^5	3.86	295 (1.04)

[a] [PFS]/[$PDMA_{54}$-CTA]/[PC] = 300:1:0.1, 10 wt % solid content.

[b] Monomer conversion determined by ^{1}H NMR.

[c] M_p and $Đ$ determined by SEC (THF solvent, PS standard).

[d] D_h and *PSD* determined by DLS.

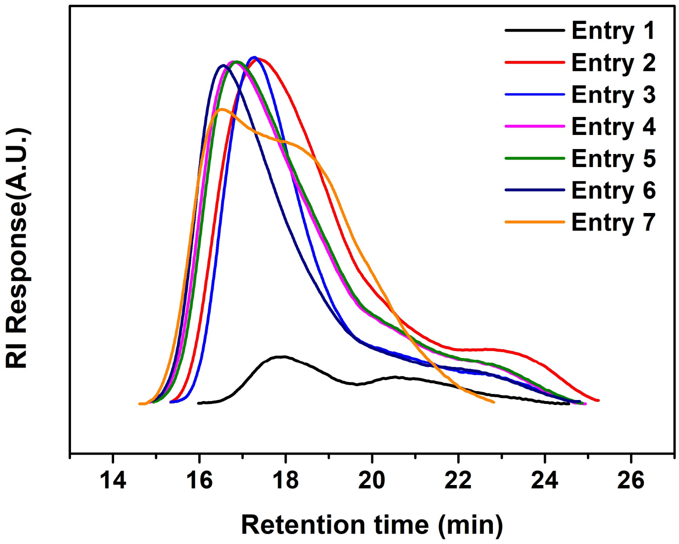

Figure S3 SEC profiles for reactions in Table S3 (light intensity = 0.56 mW/cm^2).

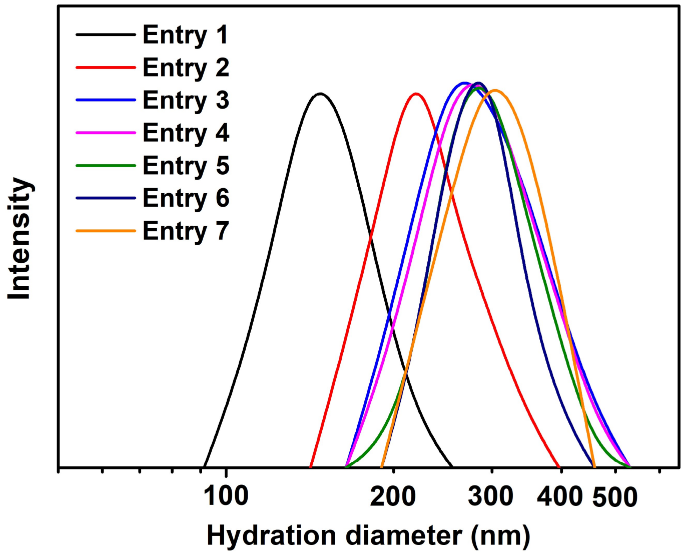

Figure S4 DLS intensity-average hydration diameter (D_h) distribution of for reactions in Table S3 (light intensity = 0.56 mW/cm^2). D_h of small particles are not detected, probably owing to their minor content and lower signal strength that have been neglected by the instrument.

Calculation for the volumes of the PPFS particles:

The volumes of PPFS particles could be calculated by Equation S1 according to volume (*V*) formula of spheroid, where *D* represents the particle diameter determined by DLS.

$$V = \frac{\pi D^3}{6} \quad (1)$$

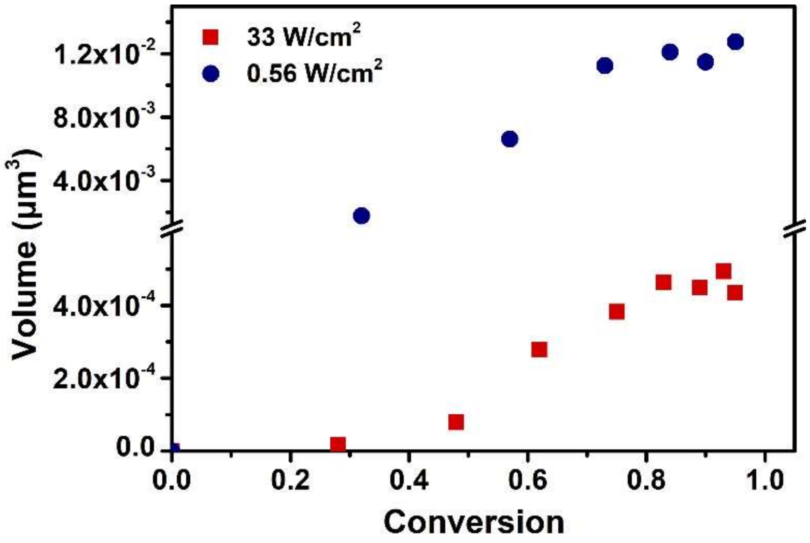

Figure S5 Evolution of particle volume (calculated based on Figure 1c in the manuscript) versus monomer conversion.

4. Preparation of PPFS particles with different fluorinated lengths

General experimental operation for Table S4:

An oven-dried 2 mL vial equipped with a stir bar was charged with 0.5 mmol monomer at corresponding molar ratio of [PFS]/[$PDMA_{54}$-CTA] (Table S4) and 1 mL DMSO. After the vial was sealed with a rubber septum, the solution was deoxygenated with three freeze-pump-thaw cycles under N_2 atmosphere. Then, the mixture was exposed to light-irradiation at 33 mW/cm^2 for 8 h while cooling with compressed air to maintain room temperature (25 °C). After reaction, NMR, DLS, SEC measurements were conducted using the reaction mixture without further purification to give summarized results in Table S4.

Table S4. Summarized results for $PDMA_{54}$-*b*-$PPFS_n$ prepared at different ratios of PFS/CTA under light intensity of 33 mW/cm^2. [a]

Entry	[PFS]/[PDMA-CTA]	Conv. [b] (%)	DP (PFS)	$M_{n, SEC}$ [c] (Da)	$M_{p, SEC}$ [c] (Da)	$M_{n, Th}$ [d] (Da)	$Đ$ [c]	D_h [e] (nm) (*PSD*)
1	100/1	> 99	100	2.31×10^4	2.43×10^4	2.49×10^4	1.20	83 (1.08)
2	200/1	> 99	200	4.23×10^4	4.41×10^4	4.41×10^4	1.36	95 (1.06)
3	300/1	> 99	300	6.59×10^4	6.91×10^4	6.33×10^4	1.51	108 (1.10)
4	500/1	> 99	500	9.84×10^4	1.23×10^5	1.02×10^5	1.63	119 (1.18)

[a] [PDMA-CTA]/[PC] = 10:1, 10 wt % solid content.

[b] Monomer conversion determined by ^{1}H NMR.

[c] M_n, M_p and $Đ$ determined by SEC (THF solvent, PS standard).

[d] Theoretical molecular weight of copolymers = (target DP × monomer conversion) × $M_{(PFS)}$ + DP × $M_{(DMA)}$ + $M_{(TTC)}$.

[e] D_h and *PSD* determined by DLS.

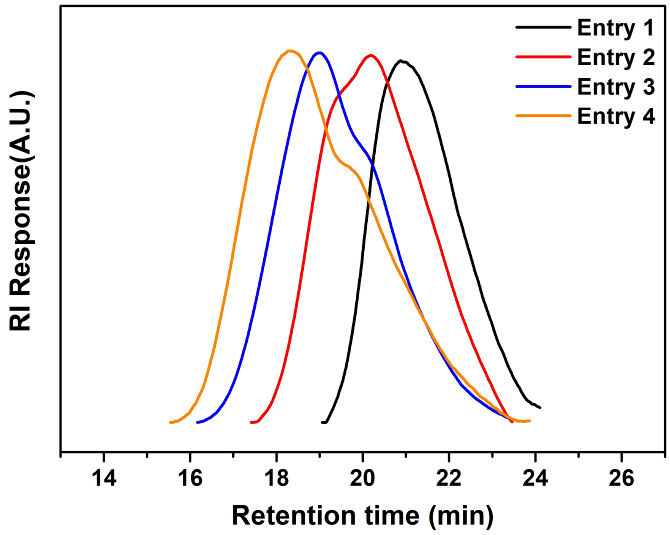

Figure S6 SEC profiles for reaction mixtures in Table S4.

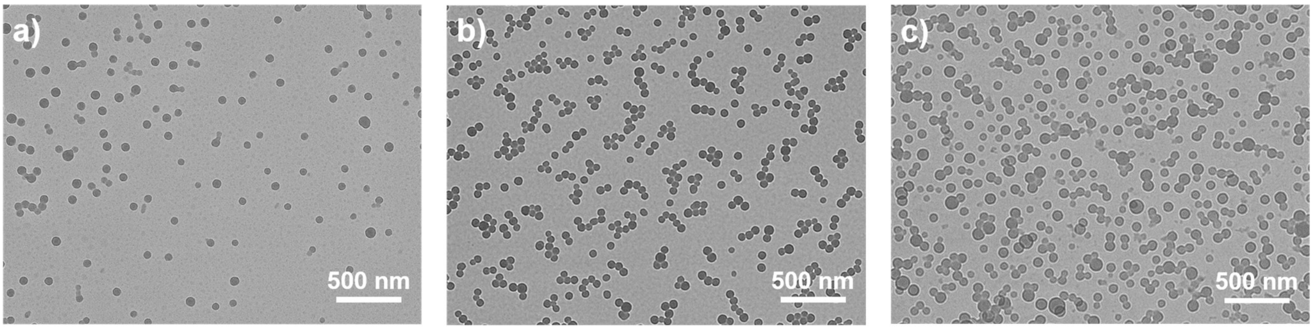

Figure S7 TEM images of $PDMA_{54}$-*b*-$PPFS_n$ nanoparticles synthesized by exposing to light irradiation of 33 mW/cm^2 (Table S4). a) $PDMA_{54}$-*b*-$PPFS_{100}$ (entry 1), b) $PDMA_{54}$-*b*-$PPFS_{200}$ (entry 2), c) $PDMA_{54}$-*b*-$PPFS_{500}$ (entry 5).

5. Investigation on the formation of RB particles

General experimental operation for Table S5:

0.5 mmol PFS, PDMA-CTA, $Ir(ppy)_3$ and 1 mL DMSO ([PFS]/[PDMA-CTA]/[PC] = 200/1/0.1) were added in an oven-dried 2 mL vial equipped with a stir bar. Then, the solution was deoxygenated with three freeze-pump-thaw cycles under N_2 atmosphere after sealing the vial with a rubber septum. The mixture was exposed to light irradiation at 0.56 mW/cm^2 for corresponding reaction times as shown in Table S5. Subsequently, the mixture was exposed to light irradiation at 33 mW/cm^2 for 4 h to achieve complete PFS conversion. After reaction, small aliquots were taken and analyzed by NMR, DLS, SEC measurements without purification to give summarized results in Table S5.

Table S5. Optimization on the irradiation time for the synthesis of RB particles. [a]

Entry	Time [b] (min)	Conv. [c] (%)	$M_{p,\ SEC}$[d] (Da)	$Đ$[d]	D_h[e] (nm) (*PSD*)
1	120	> 99	9.80×10^4	1.65	273, 70 (1.42)
2	240	> 99	4.55×10^5	3.89	321 (1.13)
3	360	> 99	6.22×10^5	4.58	330 (1.09)
4	480	> 99	9.17×10^5	6.53	341 (1.14)
5	600	> 99	9.45×10^5	6.21	335 (1.07)
6	720	> 99	9.56×10^5	5.39	327 (1.05)

[a] [PFS]/[$PDMA_{54}$-CTA]/[PC] = 300:1:0.1, 10 wt % solid content.

[b] Exposing time of weak-light irradiation.

[c] After successive irradiation using two light intensities, monomer conversions were determined by ^{1}H NMR.

[d] M_p and $Đ$ determined by SEC (THF solvent, PS standard). For bimodal SEC profiles, M_p value of the peak with a higher molecular weight is shown.

[e] D_h and *PSD* determined by DLS.

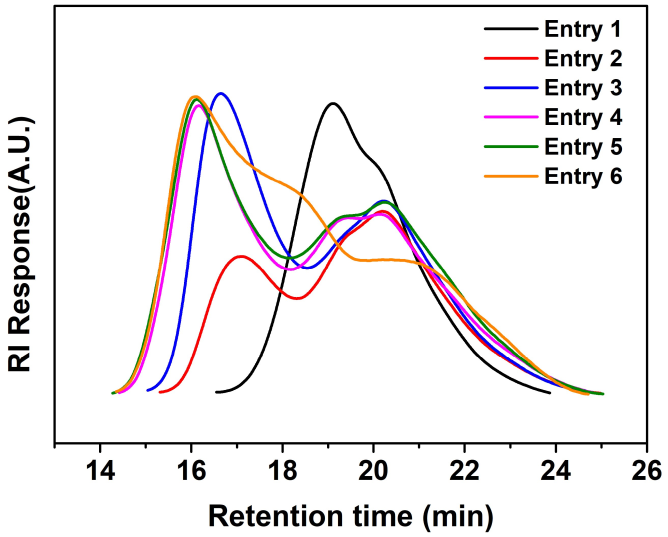

Figure S8 SEC profiles for reaction mixtures in Table S5.

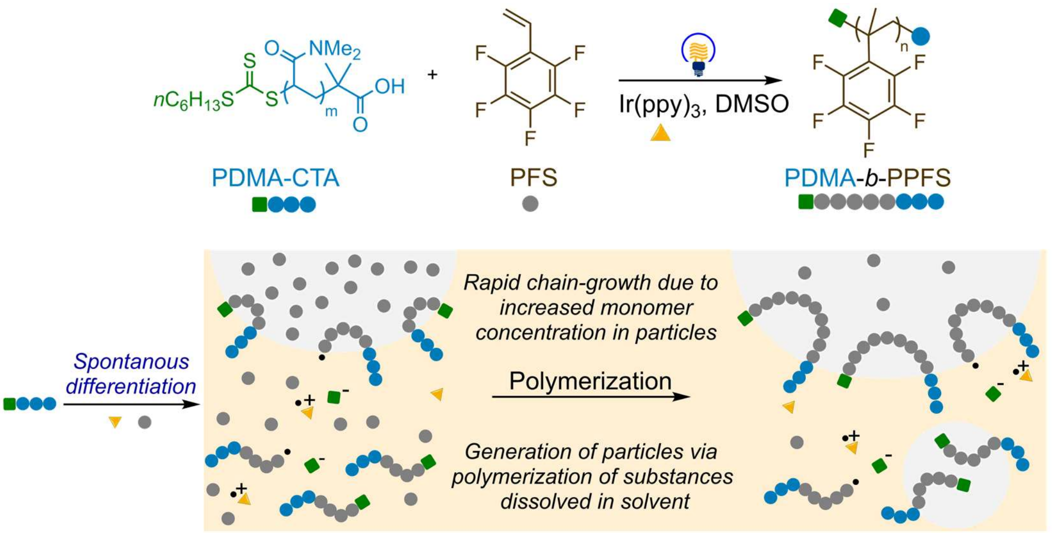

Figure S9 Hypothesized mechanism for the formation of UHMW PPFS. Due to the slow polymerization rate under weak-light irradiation, a portion of PDMA-CTA would first grow into fluorinated polymers and lead to the generation of fluorous particles via self-assembly. These particles would absorb PFS in solvent, and subsequently provide fluoropolymers with high molar mass due to the increased monomer concentration. Meanwhile, reaction between remaining PDMA-CTA and PFS dissolved in solvent would also generate new fluorous particles.

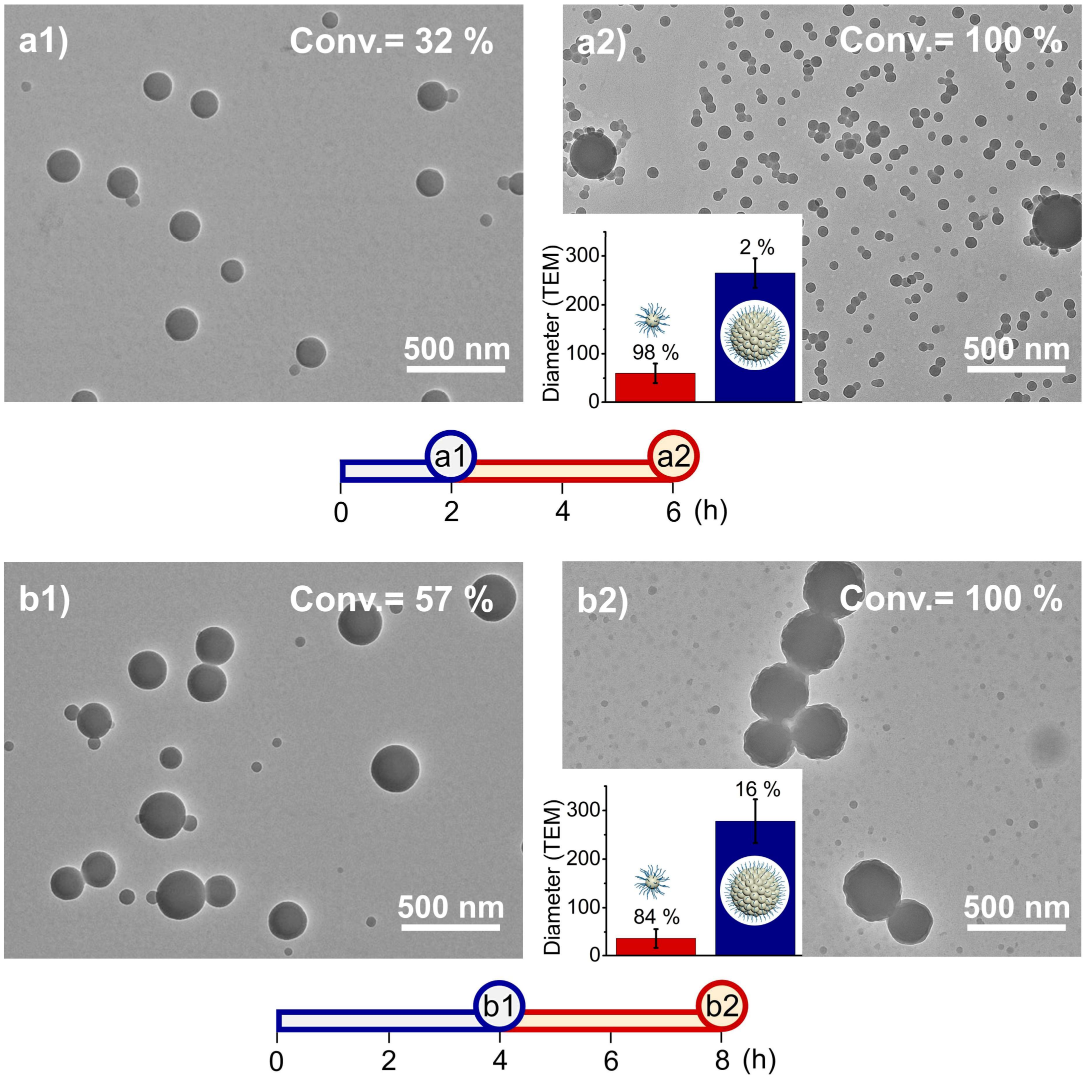

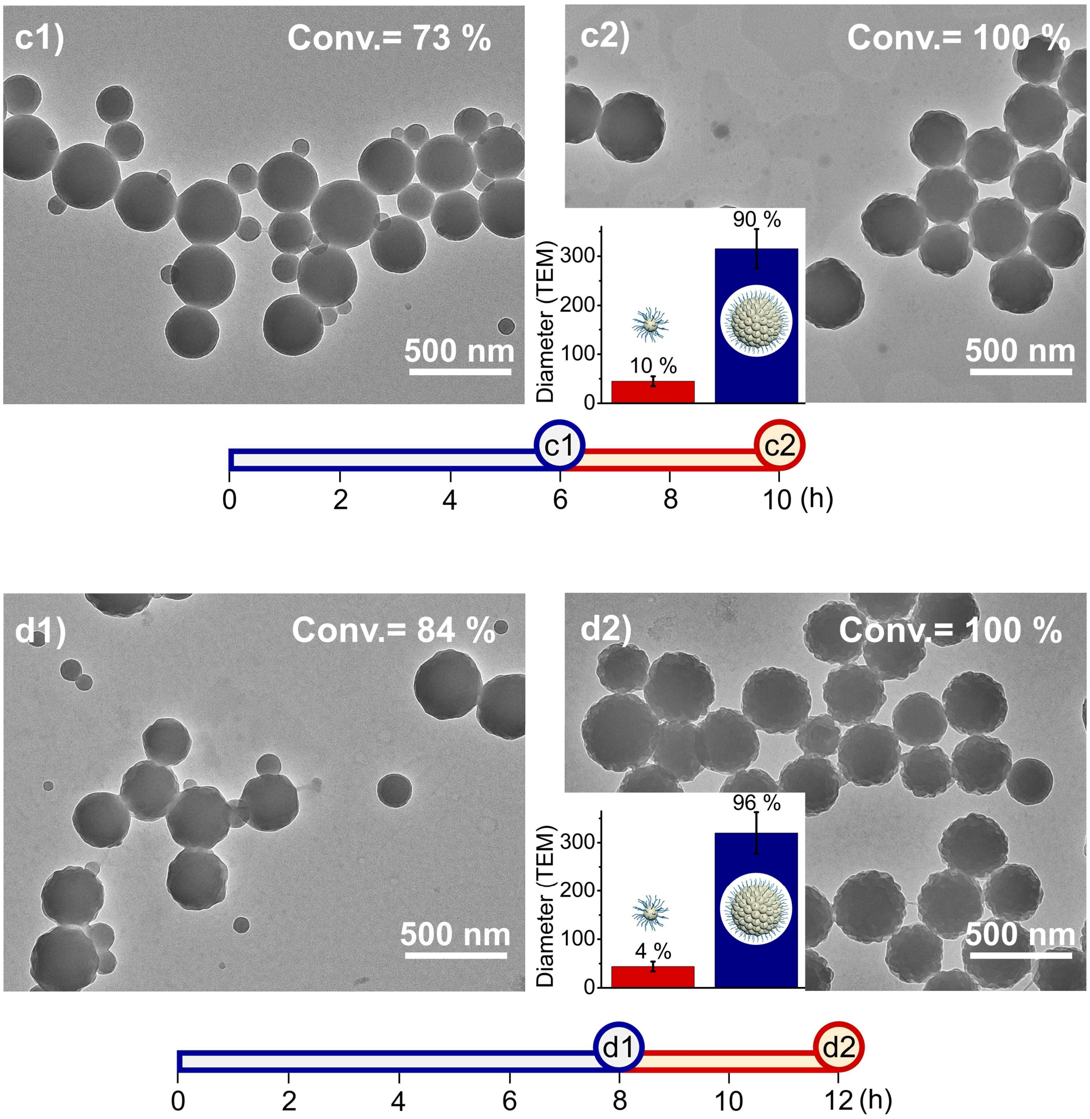

Figure S10 TEM images of fluorinated particles prepared in Table S5. a1) and a2) for entry 1; b1) and b2) for entry 2; c1) and c2) for entry 3; d1) and d2) for entry 4. For entry 5, see Figure 2a and 2b in the manuscript. For entry 6, see Figure 2c and 2d in the manuscript. The size and proportion of particles depicted in Figure S10a2, S10b2, S10c2 and S10d2 were obtained by analysing approximately 400 random particles. Bottom axes exhibit exposure times of two light intensities: blue colour for 0.56 mW/cm^2, red colour for 33 mW/cm^2.

As shown in Figure S10a1, particles of two sizes (30-60 nm and ~150 nm) were produced after exposing to weak-light irradiation for 2 h, where the large ones continued to grow into spherical particles (~280 nm, Figure S10b1 and S10c1), and evolved into larger particles with rough surface afterwards (~300 nm, Figure S10d1, and Figures 2a and 2c). When there were many monomers left before switching light intensity (i.e., monomer conversion ≤57% in Figure S10a1 and S10b1), only a small number of large particles (close to spherical shape) and lots of small nanospheres were finally obtained (Figures S10a2 and S10b2), indicating that switching to

strong-light irradiation at low to moderate PFS conversions would lead to nanoparticles with surfaces of low roughness. Based on these results, we hypothesize that as the reaction proceeds, decrease of unreacted PFS in particles would reduce the mobility of polymer chains within fused particles, which could be beneficial to the maintenance of the nonspherical morphology. Therefore, to reduce the negative influence of residual monomer, we employed strong-light irradiation to accelerate monomer consumption, and monitored the influence of switching at different times during polymerization. When light intensity was switched at 57-84% conversion of PFS (Figures S10c2 and S10d2), particles with rough surface were acquired. To give good RB morphology, 8-12 h of weak-light exposure was chosen (84-95% conversion of PFS). TEM images of 10 and 12 h of weak-light exposure are exhibited in Figure 2 of the manuscript.

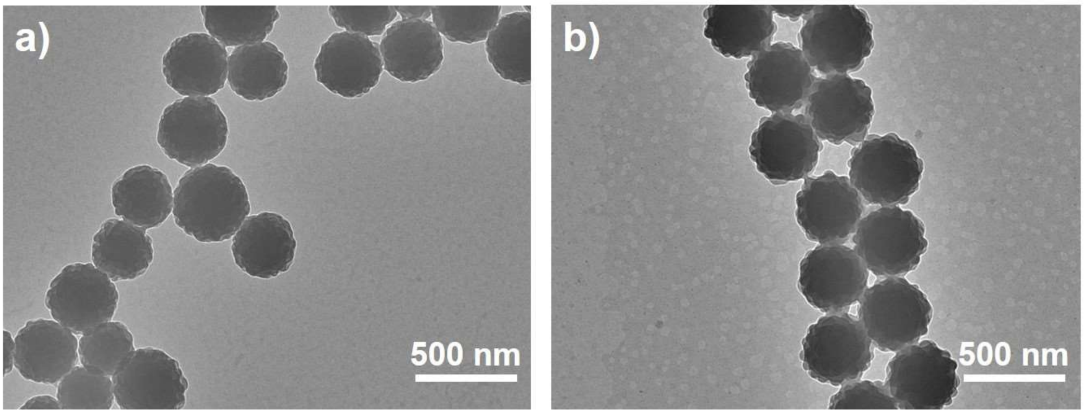

Figure S11. TEM images of particles obtained via methods **1** and **2**, respectively. a) Method **1**: 24 h weak-light irradiation (0.56 mW/cm^2). b) Method **2** (Figure 2b in the manuscript): 10 h weak-light irradiation (0.56 mW/cm^2) followed by 4 h strong-light irradiation (33 mW/cm^2).

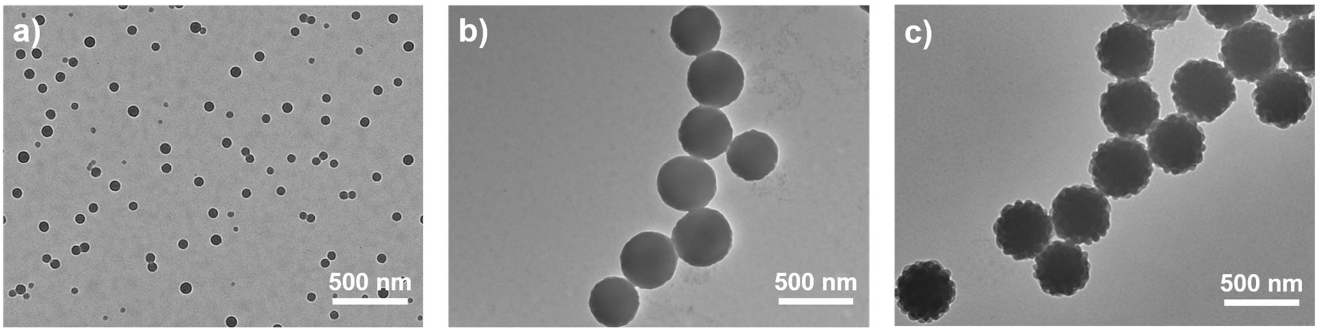

Figure S12 a) and b) TEM images of PPFS nanoparticles generated from different CTAs in Figure S13 (F-CTA and PEG_{113}-CTA, respectively). c) TEM image of PPFS nanoparticles generated from PEG_{113}-CTA using a tandem photo-irradiation.

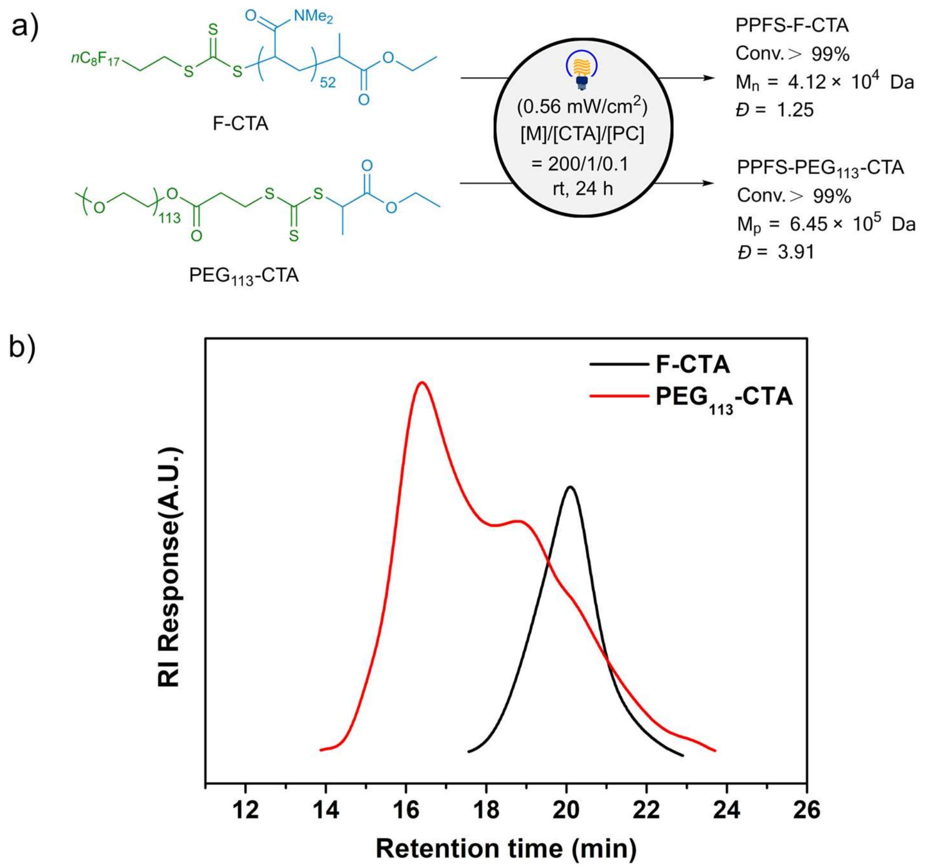

Figure S13 a) Photopolymerization of PFS using two different CTAs. b) SEC profiles.

To validate the presence of CTA-differentiation in this system,[1] we employed a new fluorinated chain-transfer agent (F-CTA, Figure S13a) in the photopolymerization of PFS. When F-CTA was used under weak-light irradiation, only small particles (Figure S12a) and polymers of symmetrical and unimodal SEC profile were generated ($Đ$ = 1.25, Figure S13b), and the $M_{n,SEC}$ is in accordance with the calculated value based on PFS conversion ($M_{n,SEC}$ = 4.12×10^4 Da, $M_{n,calc.}$ = 4.41×10^4 Da, Figures S13a and S13b). In comparison, when a polyethylene glycol-substituted CTA (PEG$_{113}$-CTA) was used under otherwise identical conditions, polymers of much higher molecular weight (M_p = 6.45×10^5 Da, Figures S13a and S13b) and particles of rough surface (Figure S12b) were generated. Using optimized conditions (8-12 h of weak-light irradiation followed by 4 h of strong-light irradiation), RB particles (Figure S12c) could be successfully obtained.

Table S6. Reaction results for the synthesis of RB particles at different [PFS]/[$PDMA_{54}$-CTA] ratios under optimized conditions.[a]

Entry	[PFS]/[$PDMA_{54}$-CTA]	Conv. [b] (%)	$M_{p, SEC}$ [c] (Da)	$Đ$ [c]	D_h [d] (nm) (*PSD*)
1	100/1	> 99	$8.57×10^5$	5.64	317 (1.13)
2	200/1	> 99	$8.96×10^5$	5.37	332 (1.07)
3	300/1	> 99	$1.03×10^6$	5.49	347 (1.07)
4	500/1	> 99	$9.41×10^5$	4.16	368 (1.05)

[a] PFS = 0.5 mmol, 1 mL DMSO solvent, successive light irradiation using two intensities (0.56 and 33 mW/cm^2)

[b] Monomer conversion determined by ^{1}H NMR.

[c] M_p and $Đ$ determined by SEC (THF solvent, PS standard). For bimodal SEC profiles, M_p value of the peak with a higher molecular weight is shown.

[d] D_h and PSD determined by DLS.

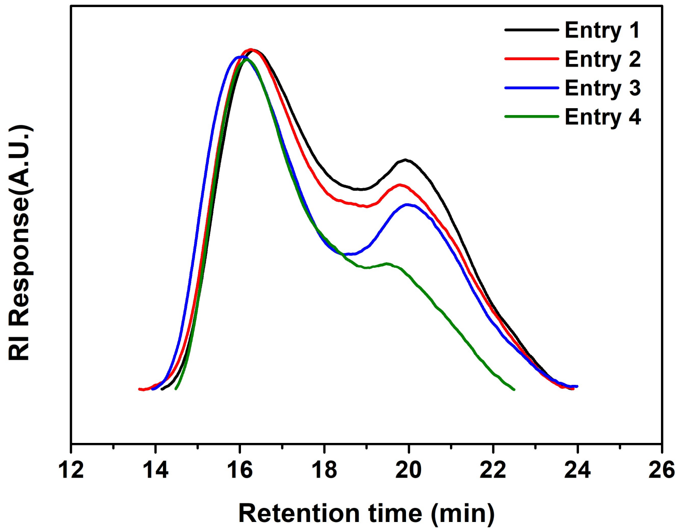

Figure S14 SEC profiles for reaction mixtures in Table S6.

Table S7. Reaction results for the synthesis of RB particles using different lengths of PDMA-CTA.[a]

Entry	CTA	Conv. [b] (%)	$M_{p, SEC}$ [c] (Da)	$Đ$ [c]	D_h [d] (nm) (*PSD*)
1	$PDMA_{28}$-CTA	> 99	$4.97×10^5$	4.09	329 (1.10)
2	$PDMA_{54}$-CTA	> 99	$8.98×10^5$	6.23	325 (1.06)
3	$PDMA_{100}$-CTA	> 99	$9.86×10^5$	5.86	276 (1.08)
4	$PDMA_{196}$-CTA	> 99	$9.12×10^5$	5.40	211 (1.04)

[a] PFS = 0.5 mmol, 1mL DMSO solvent, [PFS]/[PDMA-CTA]/[PC] = 200/1/0.1, successive light irradiation using two intensities (0.56 and 33 mW/cm^2).

[b] Monomer conversion determined by ^{1}H NMR.

[c] M_p and $Đ$ determined by SEC (THF solvent, PS standard). For bimodal SEC profiles, M_p value of the peak with a higher molecular weight is shown.

[d] D_h and *PSD* determined by DLS.

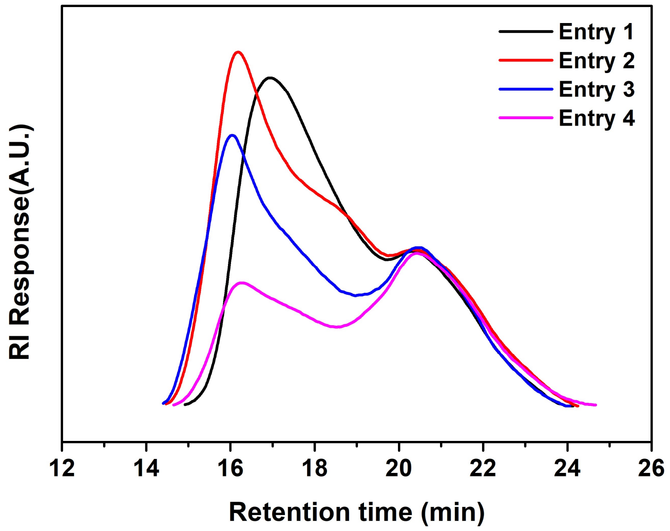

Figure S15 SEC profiles for reactions in Table S7.

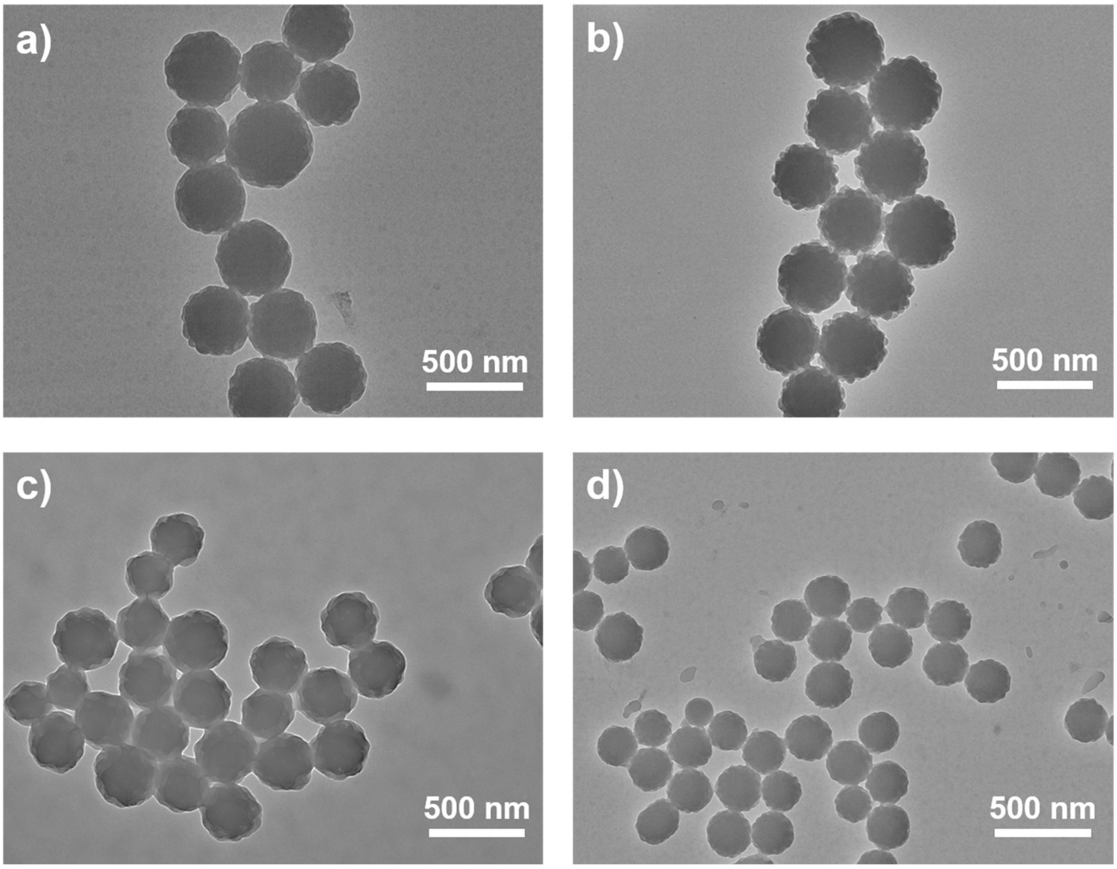

Figure S16 TEM images for RB particles prepared in Table S7. a-d) represents entries 1-4, respectively.

6. Characterization of RB particles

Table S8. The element contents of fluorinated RB particles.

Element	Line type	Standard label	Weight (%)
C	K series	C Vit	66.86
N	K series	BN	0.54
O	K series	SiO_2	2.34
F	K series	CaF_2	29.61
S	K series	FeS_2	0.65

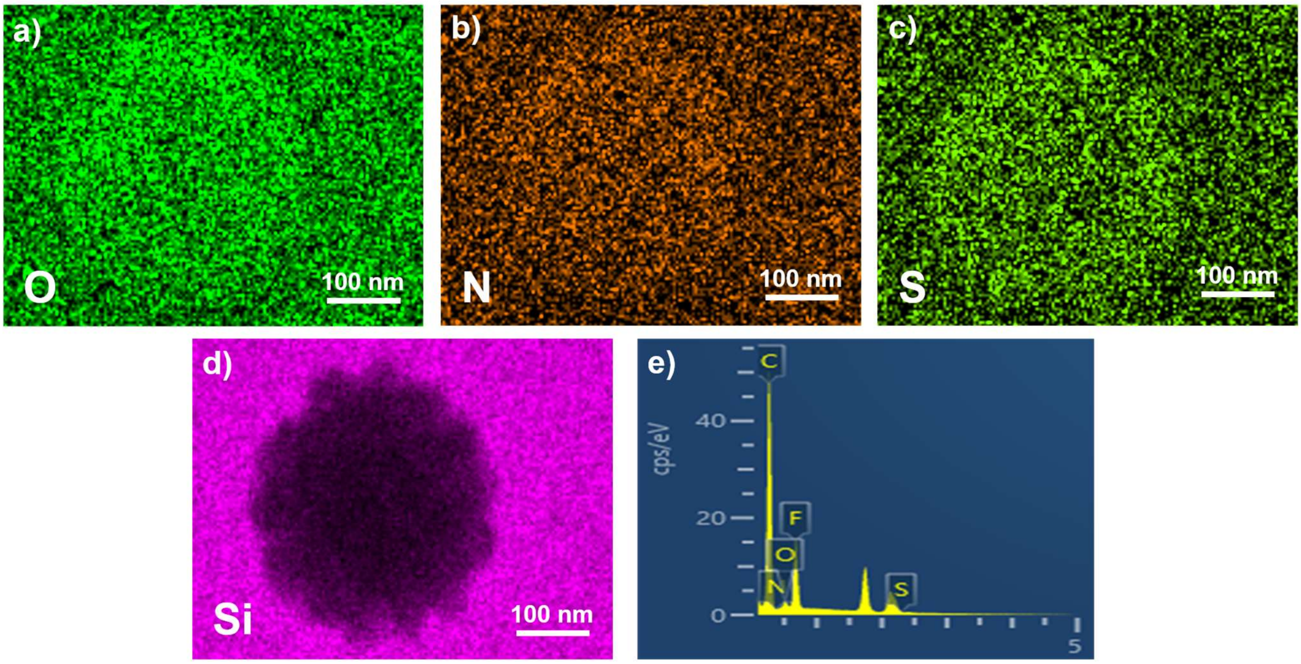

Figure S17 a-d) Mapping images of elemental O, N, S and Si, where the Si signal was attributed to the silicon substrate. e) EDS spectrum of the RB particle.

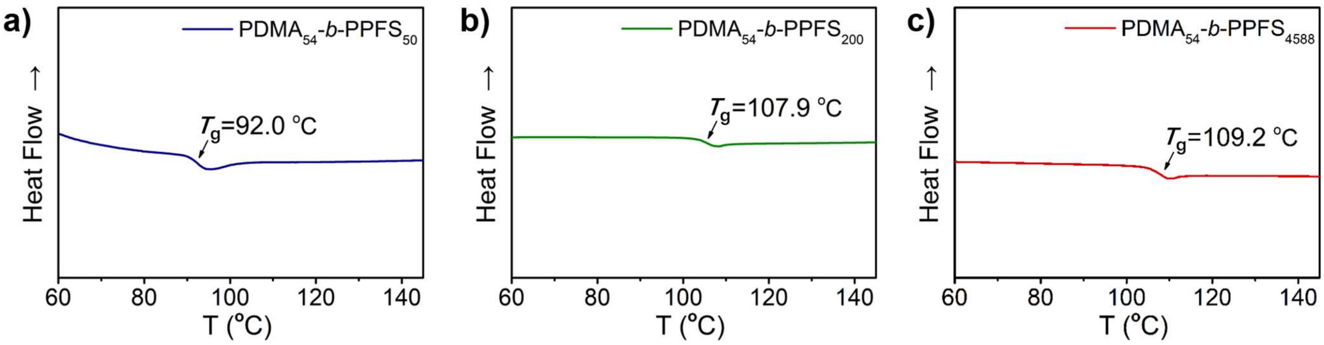

Figure S18 DSC profiles of a) PDMA$_{54}$-*b*-PPFS$_{50}$, b) PDMA$_{54}$-*b*-PPFS$_{200}$ and c) RB particles (PDMA$_{54}$-*b*-PPFS$_{4588}$).

7. Post-modification of fluorinated RB particles

General operation for the nucleophilic aromatic substitution in Figure 5:

After the preparation of RB particles (for example, entry 2, Table S6), 500 μL mixture (containing 0.25 mmol PFS functional group), was transferred into an oven-dried 1 mL vial equipped with a stir bar. Triethylamine (Et_3N) and corresponding thiol in Tables S9 and S10 ($[Et_3N]/[RSH] = 3/1$) were added into the vial. After stirring the mixture at room temperature for 24 h, small aliquots were taken and analyzed by ^{19}F NMR and HCTEM to give conversions of the $-C_6F_5$ group and TEM images, respectively. The conversion could be calculated according to previous literature (Equation S2)[2] by comparing the integration areas of para-F resonance (position p in Figure S19) with the meta'-F resonance of the products (position m' in Figure S19). In a typical ^{19}F NMR spectrum, I_1, I_2 represent the integration areas for positions of m' and p, respectively.

$$\text{Conv.}=\frac{0.5I_1}{0.5I_1+I_2} \tag{2}$$

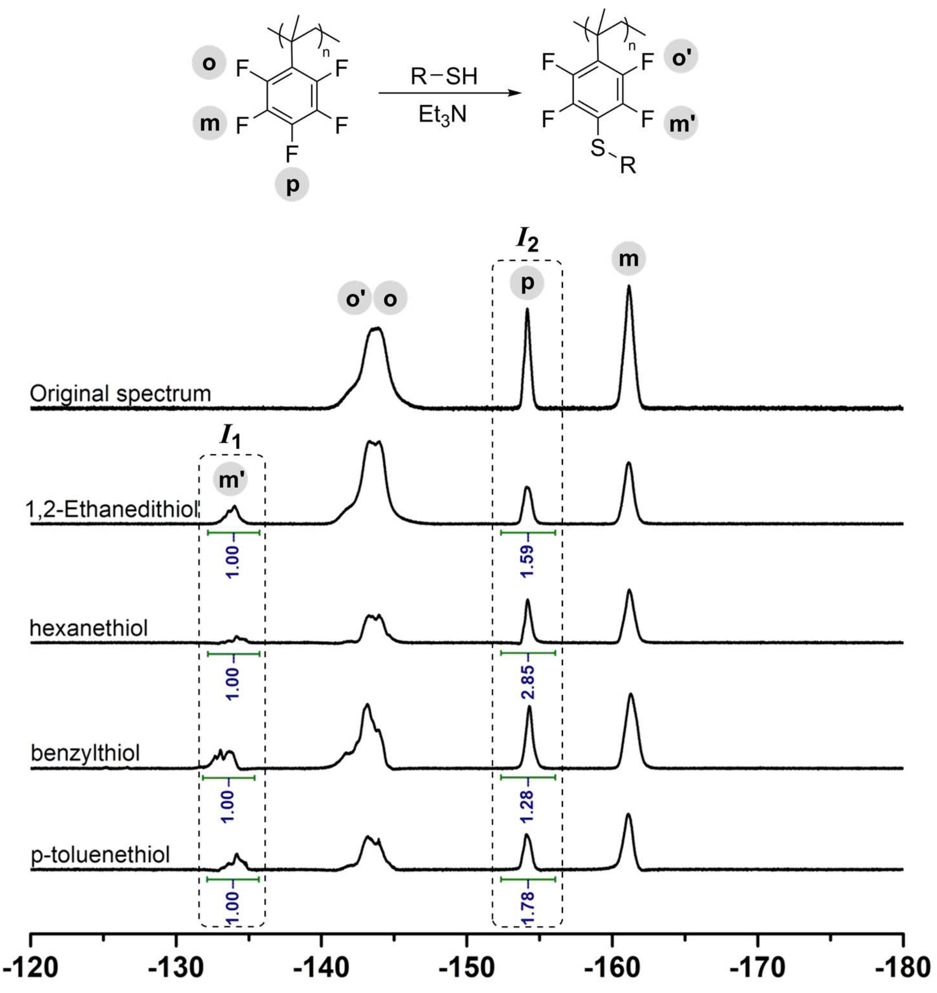

Figure S19. ^{19}F NMR spectra of original RB particles and chemically modified RB particles (Tables S9 and S10).

Table S9. Nucleophilic aromatic substitution of RB particles with 1,2-ethanedithiol.

Entry	Thiol	Feed molar ratio [PFS]/[RSH]	Conversion (%)	TEM image
1	1,2-Ethanedithiol	1/0.5	24	Figure 5b

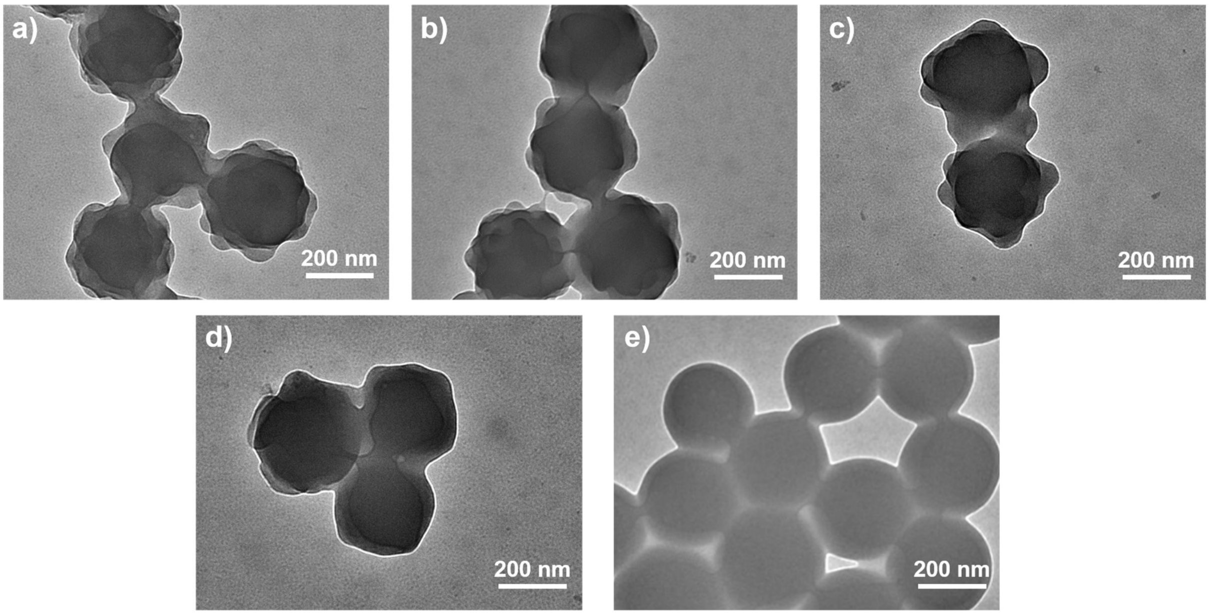

Fig S20 TEM images of crosslinked RB particles (entry 1, Table S9) treated under thermal conditions. a) 95°C; b) 105°C; c) 115°C; d) 120°C. e) TEM image of un-cross-linked RB particles treated by heating under 80°C. Operation: After reaction, the mixture of crosslinked or un-cross-linked RB particles were diluted with DMSO to 1 wt % solid content and heated under corresponding temperature for 12 h. After heating, the mixture was cooled down to room temperature and analyzed by TEM.

Table S10. Nucleophilic aromatic substitution of RB particles with different thiols.

Entry	Thiol	Molar ratio of [PFS]/[RSH]	Conversion (%)	TEM image
1	Hexanethiol	1/0.3	15	Figure S21b
2	Benzylthiol	1/0.3	28	Figure S21c
3	*p*-Toluenethiol	1/0.3	22	Figure 5c

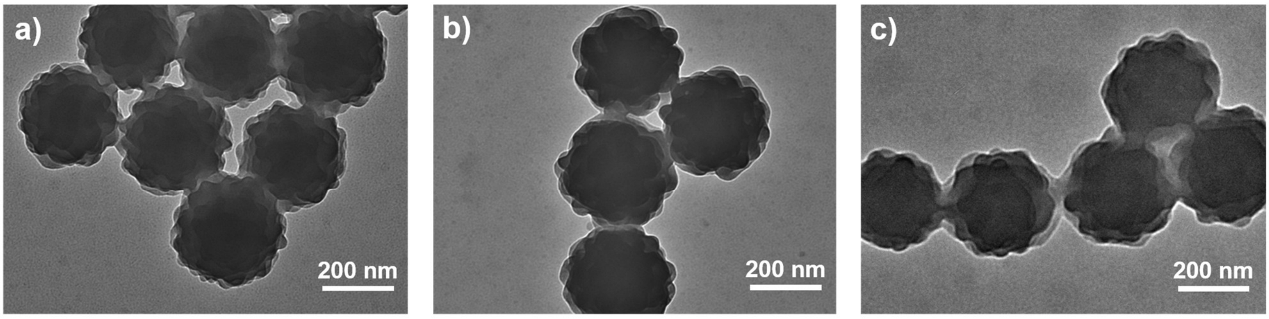

Figure S21 a) TEM image of original RB particles. b-c) TEM images of particles in entries 1 and 2 of Table S10, respectively.

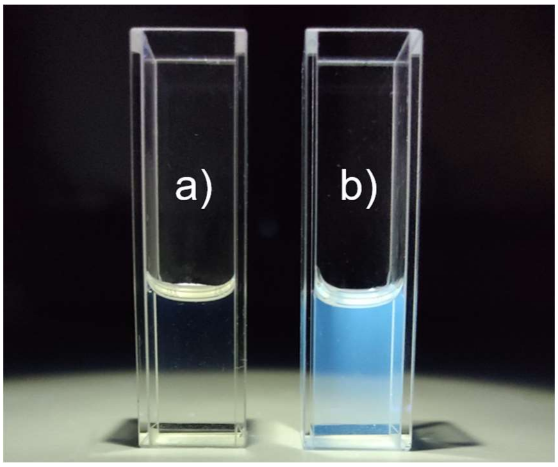

Figure S22 Optical images of a) TPE-COOH (0.5 mM in DMSO) and b) TPE-COOH (0.5 mM) and RB particles (10 wt% in DMSO) under UV irradiation (365 nm).

Section 2: Other Supplementary Information

1. Synthesis and characterization of PDMA-CTA

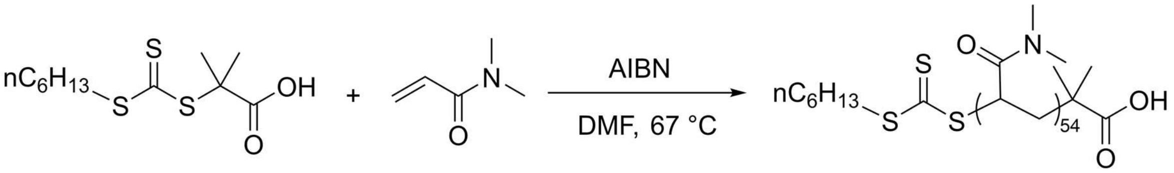

Figure S23 Synthetic route of $PDMA_{54}$-CTA.

Macromolecular initiators of PDMA-CTA were synthesized according to procedure of previous literature.[3] Taking the synthesis of $PDMA_{54}$-CTA as an example:

A 100 mL Schlenk flask equipped with a stir bar was charged with DMA (9.90 g, 100 mmol), trithiocarbonate (TTC) (280.0 mg, 1 mmol), AIBN (16.0 mg, 0.1 mmol) and DMF (50 mL). After the bottle was sealed with a rubber septum, the solution was deoxygenated for three freeze-pump-thaw cycles under N_2 atmosphere. Then, the mixture was heated at 67 °C. During reaction, small aliquots were taken via a micro-syringe under N_2 atmosphere to give monomer conversion of 54 % as analyzed by ^{1}H NMR. The reaction was quenched by exposing to air atmosphere and cooling. The mixture was added into cold ethyl ether to give PDMA-CTA as a yellow solid by centrifugation. Obtained polymers were dissolved in acetone and precipitated with cold ethyl ether for three times. Obtained polymer was further dried under vacuum at 25 °C for 8 h. For the synthesis of other PDMA-CTAs, see Table S11.

Table S11. SEC results of PDMA-CTAs.

Entry	Feed ratio (DMA/TTC)	Conv. [a] (%)	DP (DMA)	$M_{n,\,SEC}$ [b] (Da)	$M_{n,\,NMR}$ [c] (Da)	$Đ$
1	60/1	47	28	2.93×10^3	3.07×10^3	1.05
2	100/1	54	54	4.60×10^3	5.64×10^3	1.06
3	200/1	50	100	7.72×10^3	1.02×10^4	1.11
4	400/1	49	196	1.53×10^4	1.97×10^4	1.28

[a] Monomer conversion determined by ^{1}H NMR;

[b] M_n and $Đ$ determined by SEC (DMF solvent, PMMA standard).

[c] M_n calculated by ^{1}H NMR analysis: as shown in Figure S24, I_1, I_2 represent the integration areas of protons H_e and H_b respectively. The molecular weight of PDMA-CTA is calculated with Equation S3, where M_n (DMA) is the molecular weight of DMA, M_n (TTC) is the molecular weight of the trithiocarbonate.

$$M_{n,NMR}(\text{PDMA-CTA})=\frac{I_2}{6I_1}\times M_n(\text{DMA})+M_n(\text{TTC}) \quad (3)$$

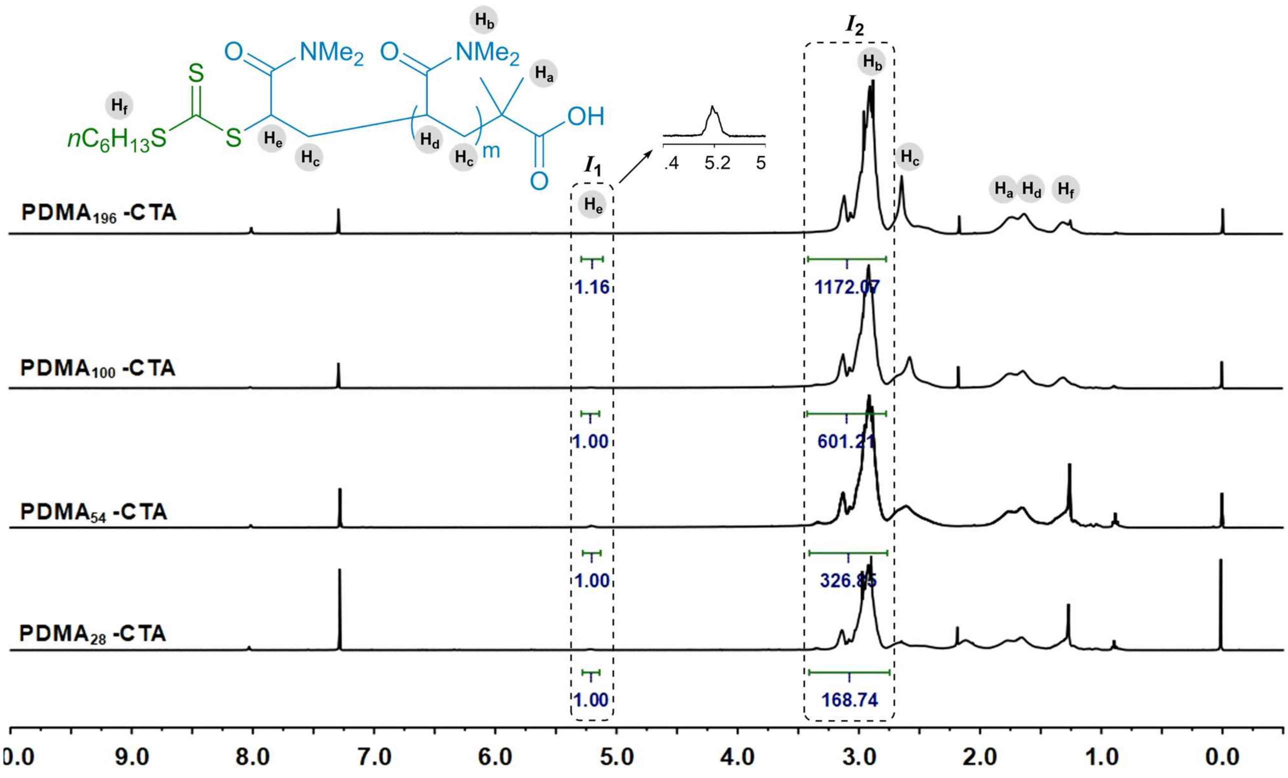

Figure S24 ^{1}H NMR spectra of PDMA-CTAs.

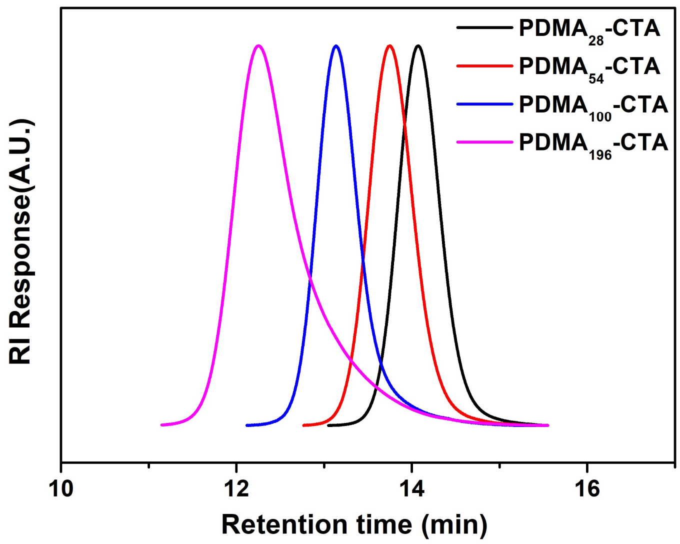

Figure S25 SEC profiles of PDMA-CTAs obtained in Table S11.

2. Synthesis and characterization of F-CTA

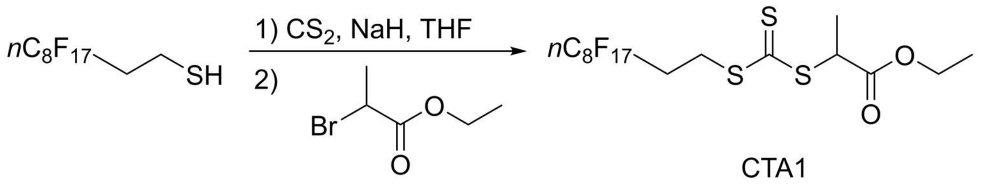

Figure S26 Synthetic route of CTA1.

CTA1 was synthesized according to previous literature.[1] 1H, 1H, 2H, 2H-perfluorodecanethiol (480.2 mg, 1.0 mmol) dissolved in 4 mL anhydrous THF was added into 100 mL flask under N_2. Then, NaH (48.4 mg, 1.0 mmol) was added into the flask at 0 °C and stirred for 15 min, CS_2 (114.2 mg, 1.5 mmol) was added dropwise into the mixture at 0 °C. After stirring the mixture at room temperature for 2 h, ethyl 2-bromopropanoate (180.6 mg, 1.0 mmol) was added into the flask at 0 °C and stirred at 25 °C for 12 h. After reaction, the crude product was extracted with EtOAc and water. The separated organic layer was dried over Na_2SO_4 and concentrated under vacuum. The product was purified by silica gel column chromatography (0-5% EtOAc in petroleum ether) to give CTA1 (531.8 mg, 81%) as a yellow solid. ^{1}H NMR (400 MHz, $CDCl_3$) δ: 4.82 (q, J = 7.2 Hz, 1 H), 4.24 (q, J = 7.2 Hz, 2 H), 3.62 – 3.58 (m, 2 H), 2.62 – 2.46 (m, 2 H), 1.63 (d, J = 7.2 Hz, 3 H), 1.29 (t, J = 6.8 Hz, 3 H) ppm; ^{13}C NMR (100 MHz, $CDCl_3$) δ: 220.4, 170.7, 119.8 – 108.1 (m, 8 C), 62.0, 48.5, 30.4 (t, J = 22.0 Hz), 27.30 (t, J = 4.0 Hz), 16.7, 13.9 ppm; ^{19}F NMR (376 MHz, $CDCl_3$) δ: -81.10 (t, J = 11.3 Hz, 3 F), -114.45 – -114.53 (m, 2 F), -121.78 – -122.14 (m, 6 F), -122.82 – -122.98 (m, 2 F), -123.41 – -123.52 (m, 2 F), -126.32 – -126.42 (m, 2 F) ppm.

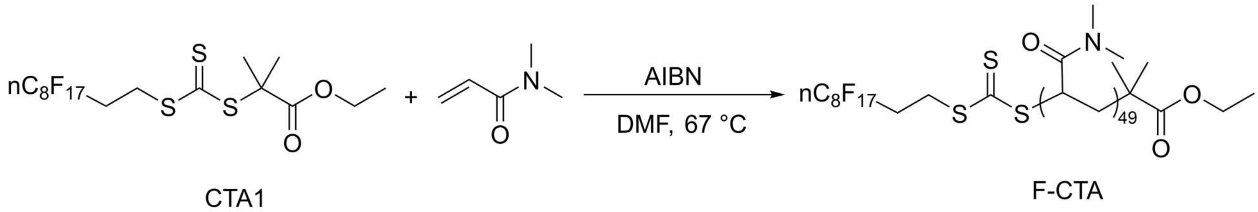

Figure S27 Synthetic route for F-CTA.

The synthesis of F-CTA is similar to PDMA-CTA. The reaction was conducted at

[DMA]/[CTA1]/[AIBN] = 100/1/0.1 in DMF. Monomer conversion of 49% was obtained as determined by ^{1}H NMR spectroscopy. After reaction, the polymerization was quenched by exposing to air. Reaction mixture was added into cold ethyl ether to give crude product as a yellow solid. The crude product was dissolved in acetone and precipitated with cold ethyl ether for three times to remove impurities. ^{1}H and ^{19}F NMR spectra of F-CTA are depicted in Figure S28 and S29.

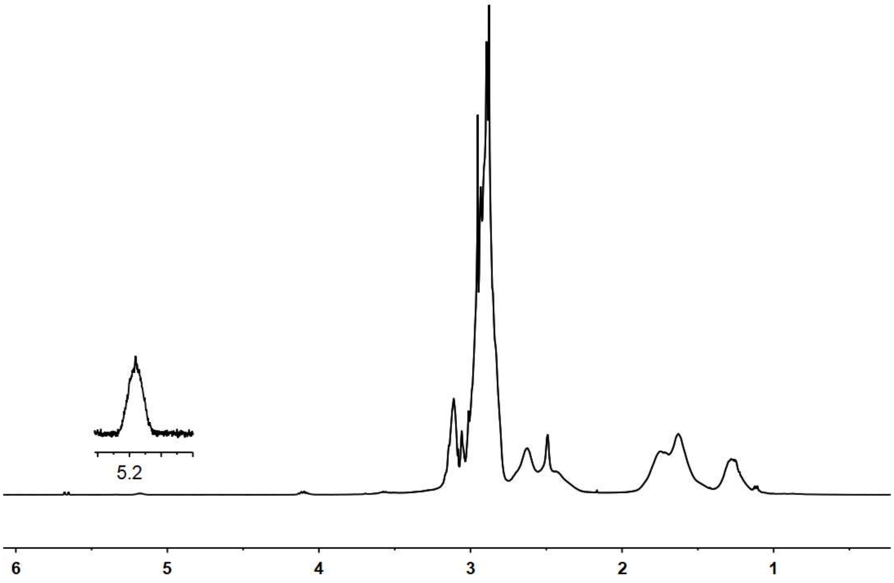

Figure S28 ^{1}H NMR spectrum of F-CTA.

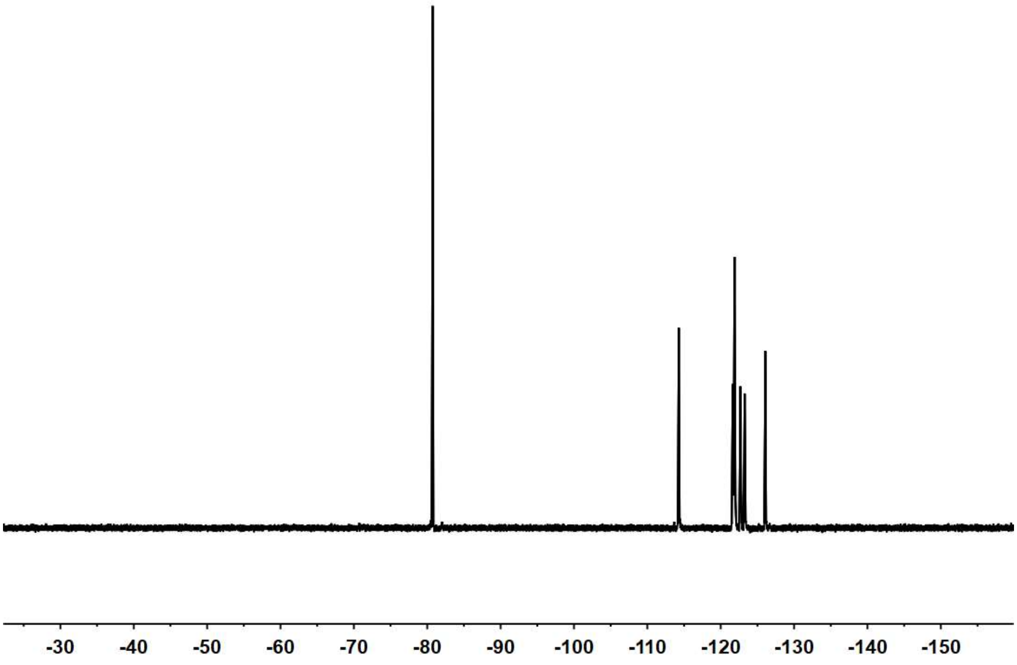

Figure S29 ^{1}F NMR spectrum of F-CTA.

3. Synthesis and characterization of PEG_{113}-CTA

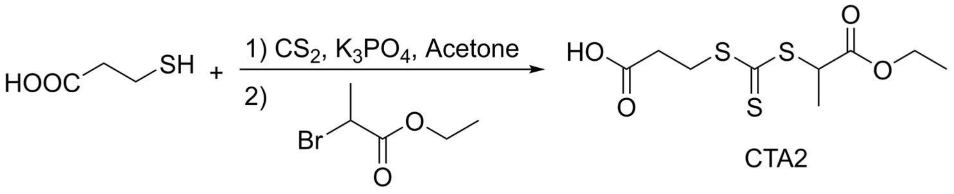

Figure S30 Synthetic route for CTA2.

CTA2 was synthesized according to previous literature.[4] A 10 mL flask equipped with a stir bar was charged with 3-mercaptopropionic acid (106.1 mg, 1 mmol), potassium phosphate (212 mg, 1 mmol) and 2 mL anhydrous acetone under N_2. After stirring for 5 min, CS_2 (137.0 mg, 1.8 mmol) was added dropwise into the flask at 0 °C. After stirring the mixture at room temperature (25°C) for 2 h, ethyl 2-bromopropanoate (199.1 mg, 1.1 mmol) was added dropwise into the flask at 0 °C. The reaction was stirred at room temperature (25°C) for 24 h. After reaction, the mixture was filtered and concentrated under vacuum. The mixture was washed with 1 M HCl aqueous solution. Separated water phase was extracted with DCM for three times. The separated organic layer was dried over Na_2SO_4 and concentrated under vacuum. Obtained residue was purified with silica gel column chromatography (0-1% EtOAc in petroleum ether) to give PEG_{113}-CTA (260.1 mg, 92%) as a yellow solid. ^{1}H NMR (400 MHz, $CDCl_3$) δ: 9.13 (br, 1 H), 4.82 - 4.78 (q, J = 7.2 Hz, 1 H), 4.21 (q, J = 6.8 Hz, 2 H), 3.61 (t, J = 6.0 Hz, 2 H), 2.83 (t, J = 4.8 Hz, 2 H), 1.61 (d, J = 7.2 Hz, 3 H), 1.28 (t, J = 7.2 Hz, 3 H) ppm.

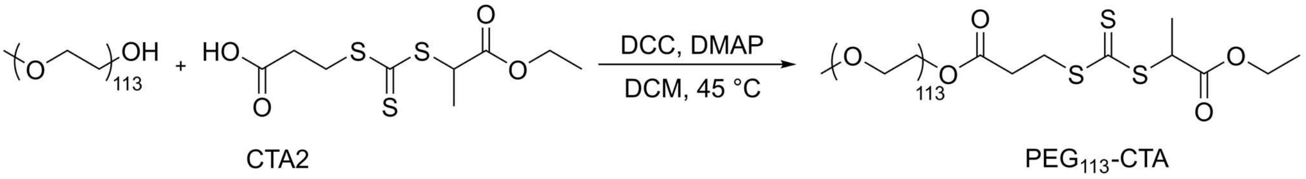

Figure S31 Synthetic route for PEG_{113}-CTA.

An oven-dried 100 mL round-bottom flask equipped with a magnetic stir bar was charged with CTA2 (2.82 g, 10 mmol,), dicyclohexylcarbodiimide (DCC) (2.07 g, 10 mmol), 4-dimethylpyridine (DMAP) (24.4 mg, 0.2 mmol) and 15 mL anhydrous CH_2Cl_2. After the flask was cooled to 0 °C, polyethylene glycol (PEG_{113}, M_n = 5.0 kDa, $Đ$ = 1.06, 5.00 g, 1 mmol) was added into the flask

under stirring. The mixture was stirred at 45°C for 3 days. After reaction, the mixture was filtered and concentrated under vacuum. Obtained solids were purified by column chromatography (50% EtOAc in petroleum ether and methanol) to give PEG_{113}-CTA (4.50 g, 90%) as a light yellow solid. ^1H NMR measurements were used to characterize PEG_{113}-CTA (Figure S32).

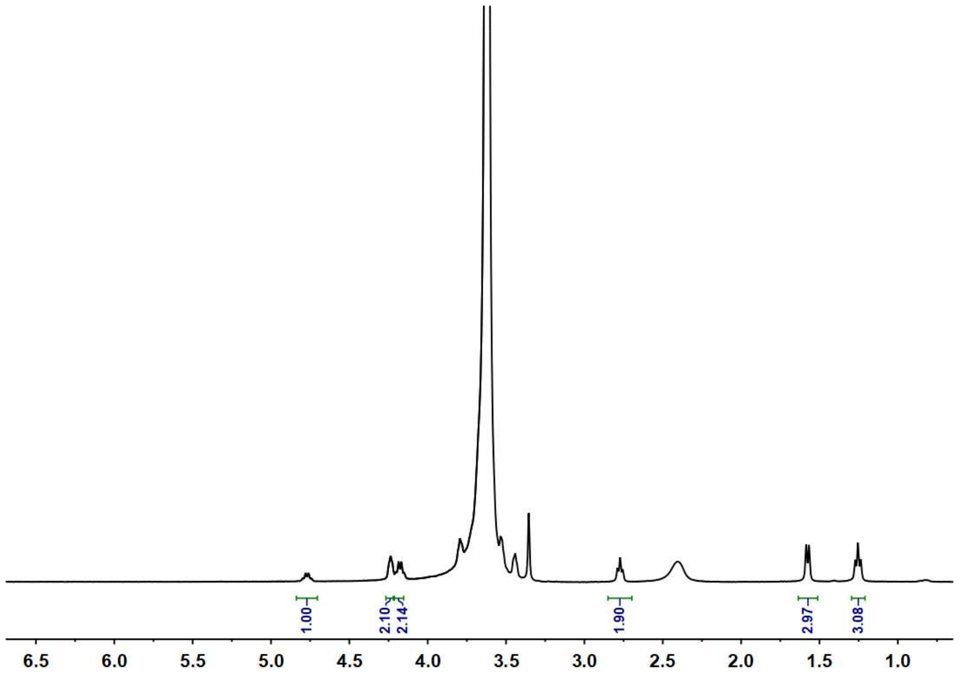

Figure S32 ^1H NMR spectrum of PEG_{113}-CTA.

4. Synthesis and characterization of TPE-COOH

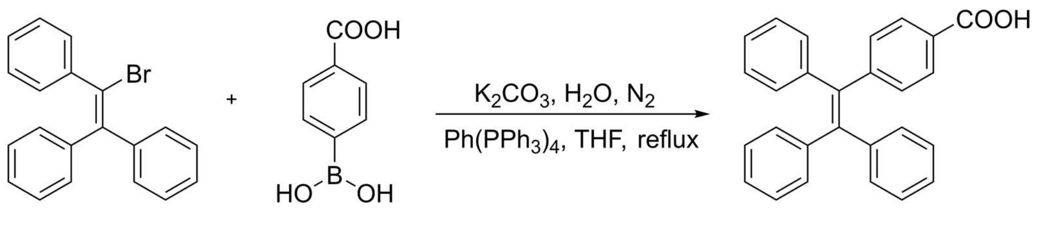

TPE-COOH

Figure S33 Synthetic route for TPE-COOH.

4-(1,2,2-triphenyl vinyl) benzoic acid (TPE-COOH) was synthesized according to literature.[5] A 100 mL round bottom flask equipped with a magnetic stir bar was charged with bromotriphenylethylene (1.68 g, 50 mmol) and 4-carboxyphenyboronic acid (1.25 g, 75 mmol), K_2CO_3 (2.07 g, 15 mmol), $Pd(PPh_3)_4$ (58.0 mg, 0.05 mmol), 30 mL THF and 5 mL H_2O under N_2 atmosphere and the mixture was stirred at 80 °C for 24 h. After reaction, the mixture was filtered

and concentrated under vacuum, the product was purified by silica gel column chromatography with ethyl acetate: petroleum ether (v: v = 1: 1) to give 4-(1,2,2-triphenyl vinyl) benzoic acid (TPE-COOH, 1.71 g, 91%) as a white power. ^{1}H NMR (400 MHz, $CDCl_3$) δ (ppm): 6.60-7.20 (m, 17H), 7.45-7.70 (s, 2H).

Reference

1. H. Gong, Y. Gu, Y. Zhao, Q. Quan, S. Han and M. Chen, *Angew. Chem. Int. Ed.*, 2020, **59**, 919-927.
2. F. Cavalli, F. R. Bloesser, C. Barner-Kowollik and L. Barner, *Chem. Eur. J.*, 2019, **25**, 10049-10053.
3. G. Moad, E. Rizzardo and S. H. Thang, *Aust. J. Chem.*, 2005, **58**, 379-410.
4. G. Mellot, P. Beaunier, J.-M. Guigner, L. Bouteiller, J. Rieger and F. Stoffelbach, *Macromol. Rapid Commun.*, 2019, **40**, 1800315.
5. H. Li, J. Chang, T. Hou and F. Li, *J .Mater. Chem. B.*, 2016, **4**, 198-201.
